# Behaviour of Extractives in Norway Spruce (*Picea abies*) Bark during Pile Storage

**DOI:** 10.3390/molecules27041186

**Published:** 2022-02-10

**Authors:** Eelis S. Halmemies, Raimo Alén, Jarkko Hellström, Otto Läspä, Juha Nurmi, Maija Hujala, Hanna E. Brännström

**Affiliations:** 1 Department of Chemistry, University of Jyväskylä, Survontie 9, 40500 Jyväskylä, Finland; raimo.j.alen@jyu.fi; 2 Natural Resources Institute Finland, Teknologiakatu 7, 67100 Kokkola, Finland; juhaottonurmi@gmail.com (J.N.); hanna.brannstrom@luke.fi (H.E.B.); 3 Natural Resources Institute Finland, Tietotie 4, 31600 Jokioinen, Finland; jarkko.hellstrom@luke.fi; 4 School of Engineering and Natural Resources, Oulu University of Applied Sciences, Yliopistonkatu 9, 90570 Oulu, Finland; otto.laspa@oamk.fi; 5 School of Business and Management, LUT University, Yliopistonkatu 34, 53850 Lappeenranta, Finland; maija.hujala@lut.fi

**Keywords:** pile storage, wood extractives, condensed tannins, stilbenes, gas chromatography with mass selective detection (GC-MS), high-performance liquid chromatography (HPLC)

## Abstract

The current practices regarding the procurement chain of forest industry sidestreams, such as conifer bark, do not always lead to optimal conditions for preserving individual chemical compounds. This study investigates the standard way of storing bark in large piles in an open area. We mainly focus on the degradation of the most essential hydrophilic and hydrophobic extractives and carbohydrates. First, two large 450 m^3^ piles of bark from Norway spruce (*Picea abies*) were formed, one of which was covered with snow. The degradation of the bark extractives was monitored for 24 weeks. Samples were taken from the middle, side and top of the pile. Each sample was extracted at 120 °C with both *n*-hexane and water, and the extracts produced were then analysed chromatographically using gas chromatography with flame ionisation or mass selective detection and high-performance liquid chromatography. The carbohydrates were next analysed using acidic hydrolysis and acidic methanolysis, followed by chromatographic separation of the monosaccharides formed and their derivatives. The results showed that the most intensive degradation occurred during the first 4 weeks of storage. The levels of hydrophilic extractives were also found to decrease drastically (69% in normal pile and 73% in snow-covered pile) during storage, whereas the decrease in hydrophobic extractives was relatively stable (15% in normal pile and 8% in snow-covered pile). The top of the piles exhibited the most significant decrease in the total level of extractives (73% in normal and snow-covered pile), whereas the bark in the middle of the pile retained the highest amount of extractives (decreased by 51% in normal pile and 47% in snow-covered pile) after 24-week storage.

## 1. Introduction

Bark contains the great majority of the hydrophilic extractives present in conifers, and it is produced as various forestry sidestreams annually on a massive scale. In 2016, the Finnish forest industry was estimated to produce 7.9 million tons of solid wood-based sidestreams [1]. Despite the high saturation of bark with potentially useful extractable chemicals for valorisation, conifer bark is still mainly used for purposes not directly related to extractives. Bark is primarily used (i) for the production of heat and energy (sometimes in a pelletised form), (ii) for non-energy purposes (e.g., roof material and mould manufacture) and (iii) for landscaping [1].

Among the various groups of bark extractives, tannins and stilbenes, which are categorised as polyphenolic and anti-oxidative compounds, are considered to be of particular interest. Generally, stilbenes (especially resveratrol) and tannins have multiple commercial applications highlighting their protective and health benefits [2,3]. Therefore, extracting these crucial compounds with suitable solvents followed by purification is considered an industrially attractive approach. However, a possible bottleneck of industrial valorisation is its logistics since high-value applications also set equally high requirements for raw materials. Therefore, it stands to reason that practices that best preserve extractives must be applied before the raw material is extracted.

In general, the storage of wood, especially pile storage, can have a considerable impact on its chemical composition [4,5,6,7,8,9]. Although pile storage of bark is a standard procedure, it may result in significant material losses, leading even to fires. However, it seems practically inevitable that some forms of raw material storage must be used, and finding a solution that does not compromise the quality of the raw material ought to be considered to be of great importance. Storing bark in an intact form on saw logs has already been discussed in previous studies [10,11]. It seems evident that such a form of storage has many advantages, as compared to pile storage, in preserving extractives in bark. This is understandable, as a smaller particle size (as in pile storage) generally exposes the chemical compounds to more degrading factors. Nevertheless, the storage of whole sawlogs may not always be feasible, and for practical reasons, some form of pile storage bark needs to be used instead. Therefore, it is necessary to understand how the pile’s internal thermokinetics affect the behaviour and degradation of extractives.

Bark extractives stored in piles are usually attacked both externally and internally [8]. Among the external factors that contribute to degradation are rain, wind and ultraviolet (UV) radiation, as well as heat, which causes evaporation [12,13,14]. On the other hand, the internal factors include bark-colonising fungi and bacteria and their enzymatic activity, as well as the self-heating of piles as a result of cellular respiration [15,16,17]. The main changes in extractives are polymerisation/depolymerisation reactions, oxidation reactions, hydrolysis reactions and phenoxy radical photo-degradation reactions [13,18]. In addition, extractives are also lost as a result of leaching (hydrophilic compounds, e.g., tannins and stilbene glycosides) and evaporation (e.g., monoterpenoids) [19,20].

While there are previous studies which aim at providing the overall picture of spruce bark, such as, the study by Krogell et al., to understand how that picture changes over time is also of key importance [21]. In this study, we evaluated the degradation behaviour of the lipophilic and hydrophilic extractives of Norway spruce (*Picea abies*) bark during pile storage over a period of 24 weeks. The main goal was to understand the speed, extent and nature of degradation and whether there is a significant difference between the sampling locations inside each pile (i.e., middle, side and top). We tested the following hypotheses: (i) the extractive content of bark stored in a pile depends on the physical location inside the pile, (ii) covering the bark pile with snow at the beginning of storage can better preserve the bark extractives and (iii) the degradation rate of extractives during pile storage is faster than that of intact bark on saw logs. Overall, the information gathered in this study facilitates the decision-making process regarding the optimisation of storage conditions for the preservation of extractives needed in the manufacture of value-added products.

## 2. Results and Discussion

### 2.1. Overview of the Change in the Chemical Composition of Bark during Storage

**Figure 1:**
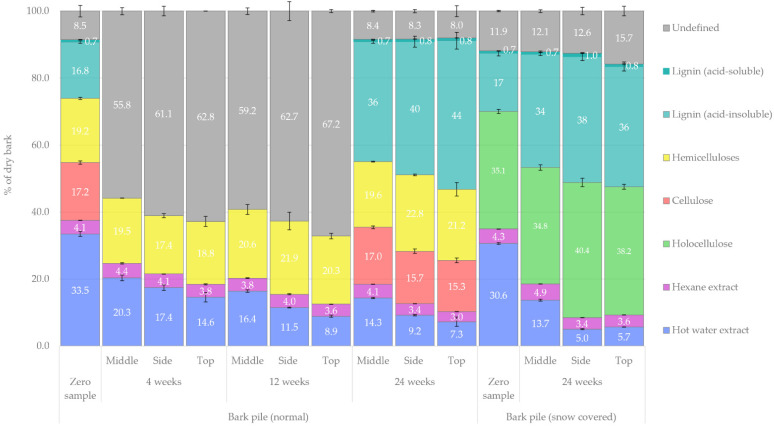
Overall changes in the bark samples’ chemical composition during storage as % of dry bark.

An overview of the changes in the chemical composition of the bark during storage is presented in Figure 1. In this figure, the gravimetrically determined amounts of total dissolved solids (TDSs) from hot-water and *n*-hexane extracts, the amount of lignin (both acid-soluble and acid-insoluble) and holocellulose as determined by acid hydrolysis and the amount of hemicelluloses and cellulose as determined by acidic methanolysis are presented. Here, the overall changes in the chemical composition are discussed with regard to the storage time, sampling location and pile covering. A more in-depth analysis of the changes within each extractive group is presented in Section 2.3. The exact values of the various compound groups, individual compounds as well as their standard deviations presented in the subsequent figures are available as Supplementary Files (link at the end of the article).

#### 2.1.1. Change in Total Dissolved Solids

##### The Effect of Storage Time

The approximate impact of storage on the relative amounts of chemical compounds in bark was as follows: over 24 weeks of storage, the amount of hydrophilic extractives decreased from 31–34% to 5–14%, the amount of lipophilic extractives changed from 4% to 3–5%, the amount of cellulose decreased slightly from 17% to 15–17%, the amount of hemicelluloses increased slightly from 19% to 20–23%, the amount of acid-insoluble lignin increased from 17% to 34–44%, the amount of acid-soluble lignin (determined by ultraviolet-visible [UV–Vis] spectrometry) increased from 0.7% to 0.7–1.0% and the amount of unidentified compounds changed from 9–12% to 8–16%. The major decrease in hydrophilic extractives agrees with previous storage studies of conifer bark. It has been previously reported that the extractives content in *Pinus sylvestris* chain flailing residue roughly halves during the first 4 weeks of storage, with the most significant changes showing in the hydrophilic fractions [22]. Similarly, Routa et al. studied *Pinus sylvestris* and *Picea abies* bark in pile storage and found that only 56% and 66% of the acetone-soluble extractives remained after eight weeks of storage, respectively [23,24]. Čabalova et al. also reported a significant decrease in *Picea abies* bark extractives extracted by ethanol-toluene mixture (2:1) and a relative increase in lignin and cellulose during 8 months of storage [25]. Compared to our previous study regarding *Picea abies* sawlog bark storage in winter and summer, the difference was noticeable. Although the initial chemical composition in the winter zero samples was very similar, the chemical composition of the 4-week stored piled bark was roughly comparable to that of 24-week stored sawlog bark [10].

Statistical tests revealed that, at the 10% level of significance, the storage time significantly affects the amounts of diterpenoids, unidentified lipophilic compounds, steryl esters, triglycerides, stilbenes, flavonoids, other phenolics, sesquistilbenes, distilbenes, unidentified hydrophilic compounds, proanthocyanidins and the TDSs of the hot-water extracts (Table 1).

Multiple different factors affect the loss of extractives during pile storage. For example, hydrophilic compounds are readily leached by moisture and rainwater, microorganisms rapidly consume some compounds (e.g., sugars), and many extractives are oxidised (e.g., resin acids) or evaporated (e.g., monoterpenoids) [5,26,27,28]. However, some extractives may be converted via heat and UV-light-induced radical chain reactions to non-extractable polymers (e.g., self-isomerisation and condensation of tannins into phlobaphenes) [20].

##### The Effect of Sampling Location

The sampling location in the pile (whether from the middle, side or top) appeared to have a systematic and predictable effect on the concentrations of bark components among all storage weeks. Statistical analysis showed that, at the 10% level of significance, the sampling location does not significantly affect the lipophilic extractives. However, a significant statistical result was obtained for the amounts of sugars and organic acids and for the ‘other hydrophilic extractives’ group (Table 1).

The degradation on the top of the pile was the most pronounced, with less degradation on the side and the most conservative degradation in the middle of the pile. These differences may largely be explained by the complex mechanics of pile storage, which differ in terms of temperature, moisture, ventilation and exposure to external forces depending on the pile formation, pile material (e.g., particle size) and the location in the pile [5,29]. The top of the pile is the part most exposed to both outside influences (e.g., wind, rain and UV light) and the pile’s internal activities (steam rising from the pile as a result of self-heating, microbial degradation). Thus, it was not surprising that the top of the pile contained a low concentration of compounds that are easily affected by these factors. Interestingly, after the initial decrease in concentration at weeks 4 and 12, certain extractive groups (e.g., sugars, sugar alcohols and organic acids) experienced an increase only in the middle point of the pile. This observation suggests that the non-volatile hydrophilic extractives from the top of the pile gradually leached downwards, creating a concentrated spot in the middle. A general trend, where the lower one goes in the pile, the higher the concentration of extractives is, could not, however, be confirmed in this study. Routa et al. also looked at the effect of location in bark pile on extractives content in *Pinus sylvestris* and *Picea abies*, but they could not find similar general trends by TDS as were found in this study [23,24]. This difference may be explained by a variety of factors, such as their choice of solvent (pure acetone), difference in extraction method, pile formation and the raw material characteristics. 

##### The Effect of Snow Cover

Minor differences were found between the results of non-covered and snow-covered bark piles. Statistical tests indicated that, at the 10% significance level, snow cover significantly affects the amounts of resin acids and unidentified lipophilic extractives, the degree of polymerisation (DP) of proanthocyanidins and the effective heating value of bark (Table 1). 

**Figure 2:**
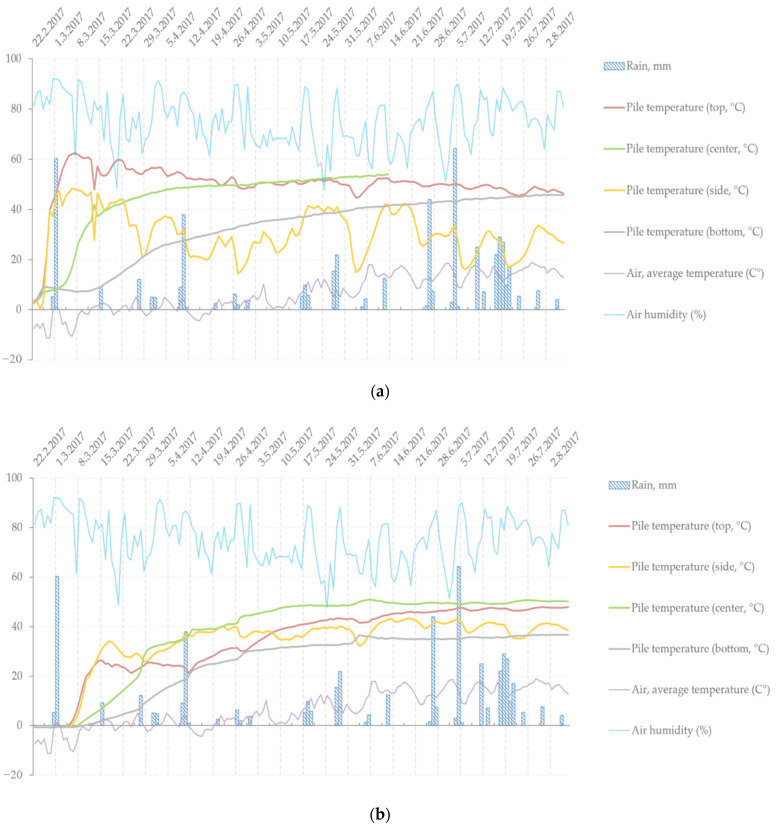
Temperature development inside the non-covered (**a**) and covered (**b**) bark piles according to the data gathered by thermocouples. The data shown are from sector one after 1 month of storage.

Notably, the concentrations of hydrophilic TDSs in the snow-covered pile were only slightly low at the beginning and end of storage compared to those in the non-covered pile. The data shown in Figure 2a,b indicate that the snow-covered pile was frozen for 10 days since the beginning of storage, unlike the non-covered pile. This means that the snow cover must have reduced the initial degradation caused by UV light and microbes. However, once the snow melted, additional slow water extraction and consequent leaching of hydrophilic extractives towards the bottom of the pile occurred. The increased moisture also enhanced the conditions for microbial invasion. Overall, although there seemed to be some initial value in covering bark piles with snow, the material losses may have been more significant in the end. Thus, it can be concluded that the hypothesis that covering bark piles with snow can help preserve the bark extractives is invalid (at least when the storage period reaches week 24). Therefore, to study the effect of snow cover on preserving extractives, sampling should be performed before the snow melts. There is evidence that semi-permeable covering of piles can reduce moisture content, temperatures and dry matter losses in forest fuel storage piles [7,9]. However, the impact of such covering during storage on extractives still needs further investigation. Recent study found that thermal drying of *Picea abies* sawmill bark in moderate temperatures will still yield major extractive losses [30].

#### 2.1.2. Changes in Carbohydrates and Lignin

Of the two studied bark piles, holocellulose was only determined from the zero samples and 24-week samples. In both piles, the holocellulose content of bark was equal at the beginning of storage (ca. 35%), and its relative proportion increased slightly towards the end of storage (because of the quicker loss of extractives). In addition, the relative total amount of lignin in bark more than doubled during storage, and the highest lignin concentrations (ca. 45%) were found at these sampling points, at which the extractive fractions were the lowest.

If no degradation occurs for hemicelluloses and cellulose, their relative proportion will increase (as in lignin). Nevertheless, the relative amounts of hemicelluloses and cellulose remained nearly the same throughout storage, indicating their slight degradation. Only on the side and top of the pile did the relative proportion between hemicelluloses and cellulose change, resulting in an overall 4% decrease in cellulose and an increase in hemicelluloses.

Similar findings of increased lignin and carbohydrate content during storage have been reported by Čabalova et al. recently [25]. However, contrary to the results presented here, the relative amount of hemicellulose was reported to have decreased while the amount of cellulose increased. This difference may be explained by the used solvent and extraction method. Compared to the unpressurised Soxhlet extraction used by Čabalova et al. [25], our hot-water extraction at 120 °C is quite harsh and may have resulted in carbohydrates that would otherwise have been included in hemicellulose and cellulose fractions to be included in the extractives fraction.

### 2.2. Biofuel Properties of Stored Bark

#### 2.2.1. Temperature Development Inside Bark Piles

The data logged from the thermocouples together with the climate conditions from a transportable weather station (air temperature, humidity and amount of rain) are displayed in Figure 2a,b. The thermocouple data revealed that the thermal activity inside the pile started almost immediately after piling the material. In general, both the centre and top of the piles experienced the highest temperatures (with a maximum at around 60 °C), whereas the side and bottom of the piles were cooler. It is also noticeable that the insides of the pile (centre and bottom) experienced a constant increase in temperature, whereas the outermost layers (top and side) experienced heavy fluctuations and correlation with rain and ambient temperature, especially on the side of the pile. Similar dependence of temperature on sampling location was also observed by Routa et al. and Krigstin et al. [23,31]. The occurrence and amount of rain was clearly most significant in June and July, towards the end of the storage period. The top of the pile was also affected by the rising steam from inside the pile. Comparing the two piles (Figure 2a,b) revealed that the snow-covered pile was initially frozen for 10 days and that the overall temperature of the pile during storage was slightly lower.

#### 2.2.2. Heating Values of Stored Bark

The heating values of the studied bark samples, their moisture and their ash, carbon, hydrogen and nitrogen contents are presented in Table 2. The results show that the average moisture content of all bark samples was approximately 57%. The sampling location also affected the moisture content of the bark. For example, in the non-covered bark pile, the moisture content was elevated to 61% at the top of the pile, remained at its original value in the middle and decreased to 41% on the side of the pile. This increased moisture on the top samples may be explained by the steam rising from inside the pile, as microbiological and chemical reactions lead to self-heating of the pile. In the snow-covered pile, presumably because of the melting of the snow cover, the 24-week samples had a high moisture content (62–70%) at all sampling locations, especially on the side and top.

The ash content of the samples underwent a gradual increase from the zero-sample level of 3.2%, especially on the side and top of the bark piles, after storage for 24 weeks, reaching peaks of 4.2% and 8.5% on the top of the non-covered and covered piles, respectively. Similar initial ash content of *Picea abies* industrial bark has been reported previously [32]. The unusually high ash content on the top of the snow-covered pile after 24 weeks of storage is most probably explained by the inorganic impurities (e.g., sand) that were mixed in with the snow that was used for covering. After the snow melted, the inorganic material accumulated on top. Moreover, the carbon content of the dry bark samples increased slightly from an initial level of 51.4% at all sampling locations, except on the top of the snow-covered pile, reaching a maximum of 52.8% at the top of the non-covered pile. The hydrogen content of the dry bark samples decreased from an initial level of 5.8% to an average of 5.6% at all sampling points, especially on the side and top of the piles and particularly in the snow-covered pile. The nitrogen content of the dry bark samples increased from an initial level of 0.47% to an average of 0.55% at all sampling points. This increase was most pronounced, especially on the side and top of the piles. However, the effective heating value remained very stable at approximately 19.3 MJ/kg at all sampling points. These heating values are slightly higher than those reported by Routa et al. for *Picea abies* bark at around 18.9 MJ/kg [24]. After storage for 24 weeks, the heating values decreased to 18.1 MJ/kg only on the top of the snow-covered pile due to increased ash content.

### 2.3. Qualitative and Quantitative Results for Bark Extracts Obtained by Gas Chromatography with a Flame Ionisation Detector/Mass Selective Detector (GC-FID/MS)

#### 2.3.1. Lipophilic and Hydrophilic Extractive Groups

**Figure 3:**
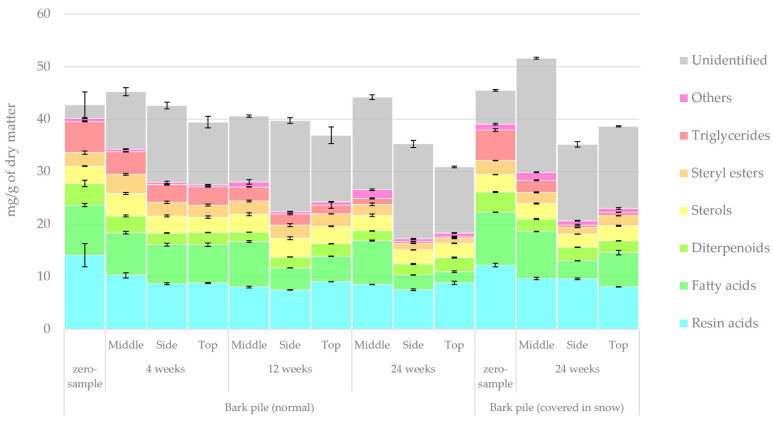
Lipophilic extractive groups in bark samples during pile storage.

**Figure 4:**
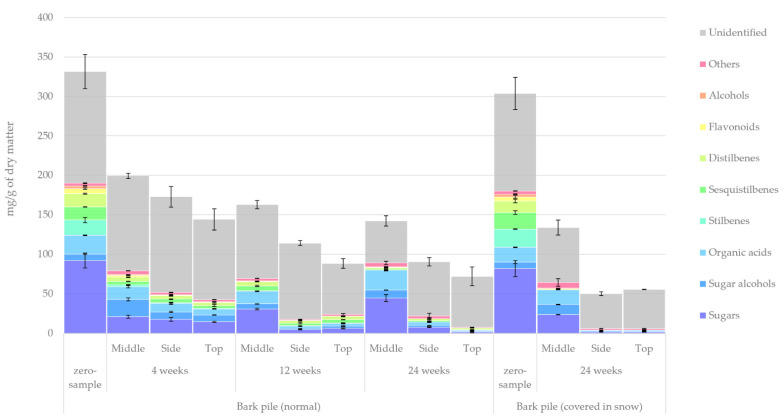
Hydrophilic extractive groups in bark samples during pile storage.

The quantified lipophilic and hydrophilic extractive groups determined using GC-FID/MS methods are presented in Figure 3 and Figure 4, respectively. The lipophilic extractives totalled 11% of all bark extractives, and their main extractive groups were resin, fatty acids, diterpenoids, sterols, steryl esters and triglycerides. In contrast, the hydrophilic extractives totalled 89% of the extractives. Their main groups were sugars, sugar alcohols, organic acids, stilbenes, sesquistilbenes and distilbenes, with the minor groups being flavonoids and other alcohols. The group defined as ‘others’ contained extractives that, despite being visible on the GC chromatograms, could not be identified or whose concentrations were very small. The ‘unidentified’ group referred to extractives that could not be detected by GC because of their low volatility or high molar weight. The relative amount of unidentified compounds increased during storage, suggesting an increase in polymerisation reactions.

**Figure 5:**
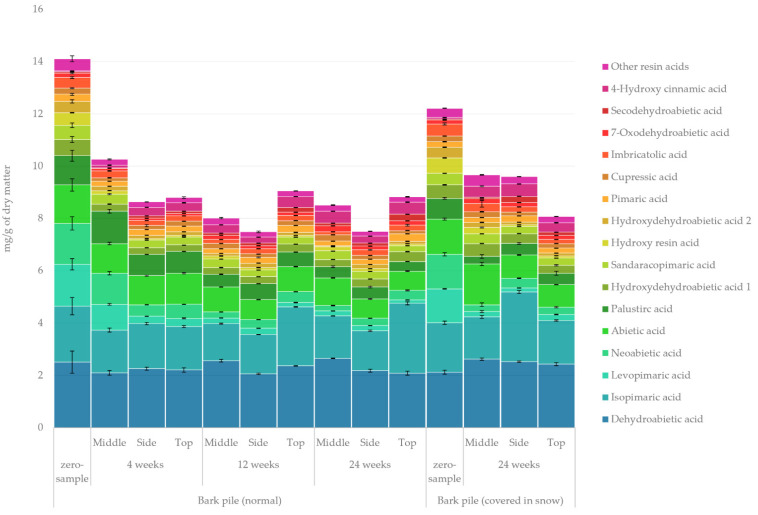
Quantified amounts of individual resin acids in the lipophilic extracts of stored bark.

**Figure 6:**
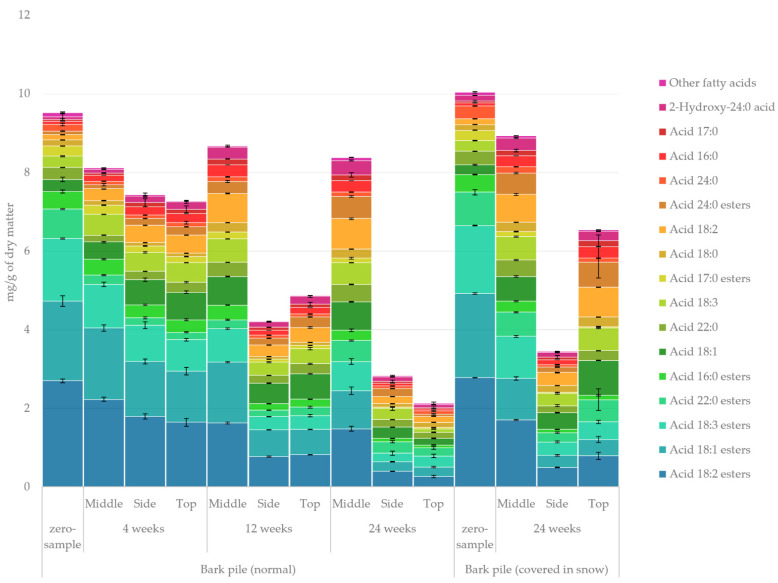
Quantified amounts of individual fatty acids in the lipophilic extracts of stored bark.

**Figure 7:**
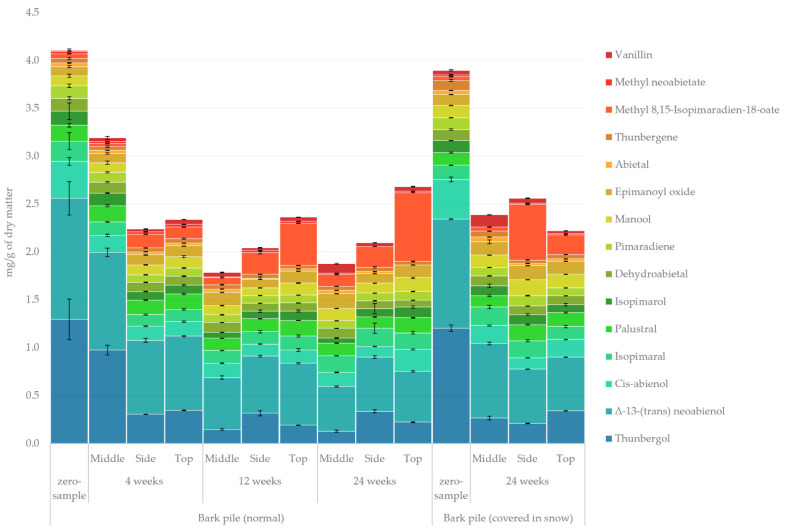
Quantified amounts of individual diterpenoids in the lipophilic extracts of the stored bark.

**Figure 8:**
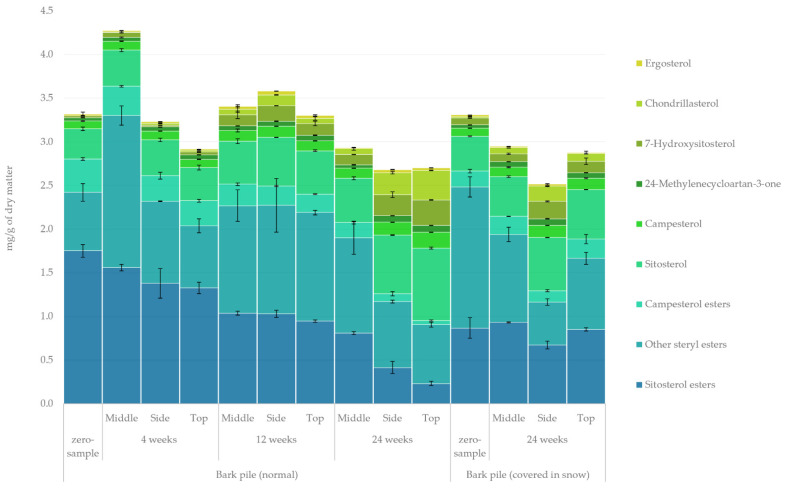
Quantified amounts of sterols and steryl esters in the lipophilic extracts of stored bark.

As shown in Figure 3, overall, there was only a slight decrease in the total amount of lipophilic extractives over a storage period of 24 weeks. The most notable changes in the chemical composition of the lipophilic extract were as follows: a decrease in resin acids from 33% to 23%, a decrease in fatty acids from 22% to 12%, a decrease in triglycerides from 14% to 2% and an increase in unidentified compounds from 6% to 44%. Thus, the results suggest that the storage of bark increases the polymerisation reactions of lipophilic compounds. The results indicate that the rate of degradation gradually slowed as the storage progressed. The overall increase in new unidentified compounds was 2.5 mg/g/storage week after 4 weeks of storage and slowed down to 0.2 mg/g/storage week after 12 and 24 weeks of storage. The concentration of lipophilic extractives decreased on the top and side of the bark pile and increased in the middle of the pile. This finding was confirmed by comparing the results obtained on week 12 and week 24 for the zero sample of the non-covered pile and the 24-week sample of the covered pile. For a more detailed analysis of the degradation pattern of individual lipophilic compounds, see Figure 5, Figure 6, Figure 7 and Figure 8. The results from our previous sawlog bark study indicate that there is much variation between individual sawlog barks, particularly in the amount of lipophilic extractives, sometimes reaching even above 70 mg/g of dry matter [10].

**Figure 9:**
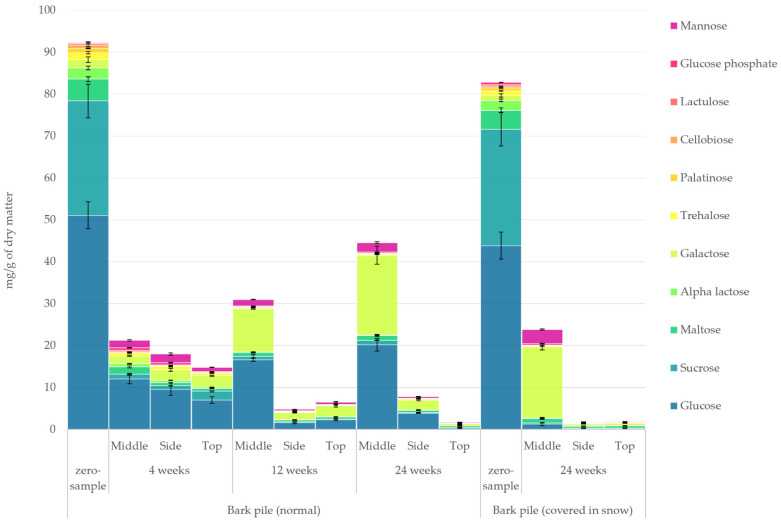
Quantified amounts of mono- and disaccharides in the hydrophilic extracts of stored bark.

**Figure 10:**
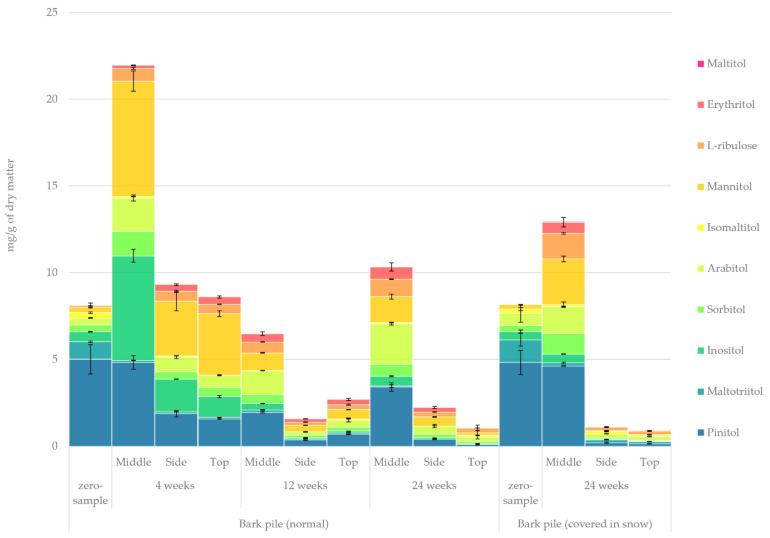
Quantified amounts of individual sugar alcohols in the hydrophilic extracts of stored bark.

**Figure 11:**
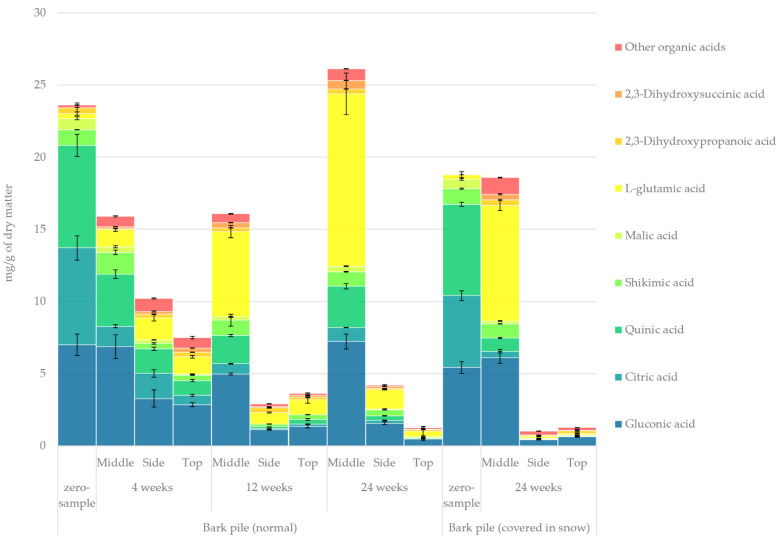
Quantified amounts of individual organic acids in the hydrophilic extracts of stored bark.

**Figure 12:**
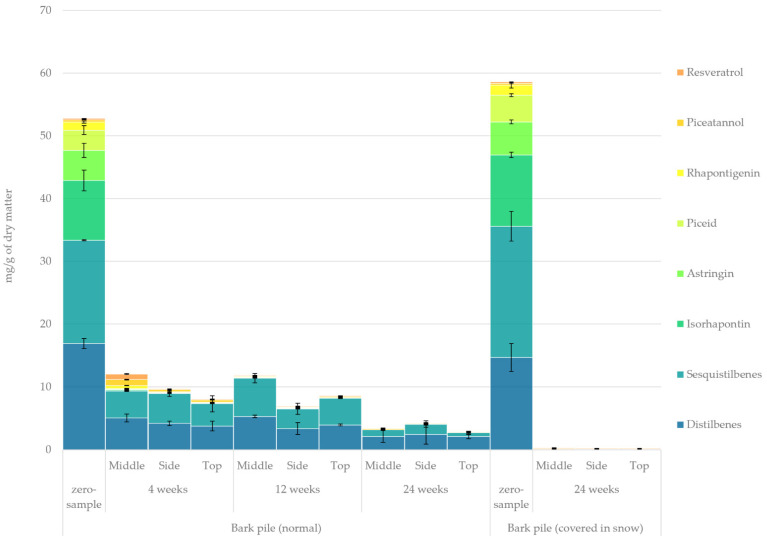
Quantified amounts of stilbenoids in the hydrophilic extracts of stored bark.

**Figure 13:**
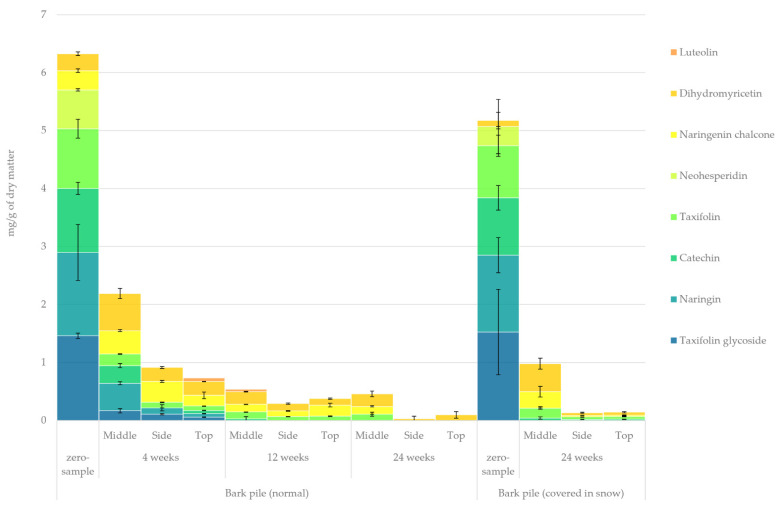
Quantified amounts of individual flavonoids in the hydrophilic extracts of stored bark.

The results outlined in Figure 4 show a clear and gradual change in the total amount of hydrophilic extractives and a dramatic decrease in the concentration of many hydrophilic extractive groups in bark resulting from pile storage. The unidentified bark extractives composed of polymeric compounds, such as condensed tannins (CTs) and oligo- and polymeric sugars, represented almost half of all hydrophilic extractives. Mono- and disaccharides represented the second-largest extractive group. The most significant changes in the relative proportion of extractives in the hydrophilic water extracts (zero sample vs. 24-week sample) were as follows: a decrease in sugars from 28% to 17% and an increase in unidentified compounds from 42% to 61%. Stilbenes, sesquistilbenes, distilbenes, flavonoids and other phenolics also experienced a major decrease in concentration, but this did not affect the total extract amount as much. Unlike with the lipophilic extractives, the relative increase in unidentified compounds seemed to result from the decrease in other compounds and not from the increase in polymerisation. For a more in-depth analysis of the hydrophilic extractive groups, see Figure 9, Figure 10, Figure 11, Figure 12 and Figure 13. A major difference is seen here to sawlog bark, where the concentration of hydrophilics remained at the level of 300 mg/g of dry bark for up to 12 weeks of winter storage [10]. This amounted to approximately 59% less hydrophilic extractives in pile-stored bark at week 12, most likely due to microbial degradation.

#### 2.3.2. Resin Acids

The quantified amount of resin acids in the lipophilic bark extracts determined using GC-FID/MS is presented in Figure 5. The results demonstrate a considerable overall decrease in the amount of resin acids during pile storage over the first 4 weeks of storage. After this initial decrease, the total amount of resin acids did not change much, and there was no apparent trend with sampling location. The general stability of resin acids has also been reported previously [10,33]. The most remarkable changes in the relative proportion of resin acids (zero sample vs. 24-week sample) were the increase in dehydroabietic acid from 18% to 28% and in isopimaric acid from 15% to 23% and the decrease in levopimaric acid from 11% to 2% and in neoabietic acid from 11% to 3%. The absolute values of the most prominent resin acids, namely dehydroabietic and isopimaric acid, remained more or less constant throughout storage. Although some reports indicate that certain fungi can reduce the amount of resin acids markedly, the way in which the degradation of resin acids halted after 4 weeks suggests that the initial drop correlated instead with the increased pile temperature [34]. This is also supported by the decrease in neoabietic and levopimaric acids, which are the most prone to thermal oxidation, Diels–Alder reaction, isomerisation and radical reactions because of their conjugated double-bond structure.

#### 2.3.3. Fatty Acids

The quantified amount of fatty acids in the lipophilic bark extracts determined using GC-FID/MS is presented in Figure 6. The changes in triglycerides and fatty acids during storage in many raw materials have been known for a long time. Fatty acids can react either by their conjugated double bonds or carboxylic acid group, leading to various different derivatives [35]. The hydrolysis of triglycerides and consequent polymerisation of the released fatty acids was reported by Ekman among the major chemical changes in wood material during storage [36]. Similarly, Nielsen et al. attributed the decrease in fatty acids during the storage of softwood chips and sawdust to polymerisation and oxidation reactions [37]. It is noteworthy that the total amount of fatty acids dropped considerably during storage, especially on the top and side of the pile, whereas the fatty acids in the middle of the pile on the other hand appeared to be remarkably well-shielded from degradation (although a change in chemical composition was observed). This clearly indicates that the degradation is connected with hydrolysation and oxidation reactions caused by external influences. Esterified fatty acids constituted the vast majority (83%) of total fatty acids at the beginning of storage. The most significant changes (zero sample vs. 24-week sample) in the relative amount of fatty acids were a decrease in fatty acid esters 18:1, 18:2 and 18:3 from 21% to 11%, from 28% to 16% and from 17% to 9%, respectively, and an increase in acids 18:1 and 18:2 and esters of acid 24:0 from 3% to 9%, from 1% to 8% and from 1% to 6%, respectively. From this, the conversion of esterified fatty acids into non-esterified fatty acids seems evident. It should be considered that the degradation of triglycerides during storage (shown in Figure 3) also releases free fatty acids. Routa et al. reported fast degradation of triglycerides during the storage of Scots pine bark, which seemingly led to an increase in the total amount of fatty acids during storage [23].

#### 2.3.4. Diterpenoids

The quantified amount of diterpenoids in the lipophilic bark extracts determined using GC-FID/MS is presented in Figure 7. The amount of diterpenoids at the beginning of storage was slightly above the levels reported by Krogell et al. (0.7 mg/g and 3.2 mg/g in inner and outer bark, respectively) [21]. A considerable overall decrease in diterpenoids was observed during the 24-week storage. Thunbergol, which is associated with anti-fungal, anti-oxidative and anti-tumour activities, was the primary diterpenoid with Δ^13^-(*trans*)neoabienol [38,39]. The most prominent changes (zero sample vs. 24-week sample) in the relative amount of diterpenoids were an increase in methyl 8,15-isopimaradien-18-oate from 1% to 16% and a decrease in thunbergol and Δ^13^-(*trans*)neoabienol from 32% to 10% and from 31% to 24%, respectively. That methyl 8,15-isopimaradien-18-oate was formed primarily on the side and at the top of the piles indicates a formation through oxidation reaction. Nielsen et al. also reported that diterpenoid degradation is affected by oxidation and polymerisation reactions [37]. Thunbergol loss was expected because it is also entirely lost during tall oil distillation [40]. Our previous study regarding sawlog bark also indicated a loss of thunbergol with the increase in ambient temperature [10]. 

#### 2.3.5. Sterols

The quantified amount of sterols and steryl esters in the lipophilic bark extracts determined using GC-FID/MS is presented in Figure 8. The major sterol in *Picea abies* is *β*-sitosterol, a prominent antibacterial and antioxidant agent [41]. The total amount of sterols ranged between 3.2–4.8 mg/g of dry matter and only a slight overall decrease was observed. Routa et al. reported similar sterol levels and only slight degradation during 8 weeks of Scots pine storage [23]. Assarson had reported similar resistance to degradation in unsaponifiable compounds (including sterols) in *Picea abies* chip pile storage [42]. The most prominent changes in the relative amount of sterols (zero sample vs. 24-week sample) were a decrease in the esters of sitosterol and campesterol from 53% to 17% and from 12% to 4%, respectively, and an increase in sitosterol, chondrillasterol and 7-hydroxysitosterol from 10% to 24%, from 0% to 8% and from 1% to 8%, respectively. Given these results, it seems that esterified sterols underwent gradual conversion into free sterols during storage. In addition, ergosterol, chondrillasterol and 7-hydroxysterol were formed as a result of storage, especially on the side and at the top of the pile, again indicating a formation through oxidation reactions [43].

#### 2.3.6. Sugars

The quantified amount of simple sugars in the hydrophilic bark extracts determined using GC-FID/MS is presented in Figure 9. Mono- and disaccharides underwent major degradation during pile storage, with only approximately 20% of the sugars remaining after storage for 24 weeks. The sampling location resulted in an increasingly greater difference in the concentration of sugars. At the end of storage, the concentration of sugars at the top of the pile decreased to vanishingly low levels, with the concentration at the side of the pile being only slightly higher. The middle of the pile, on the other hand, exhibited an increased concentration after the initial decrease at week 4. The most significant changes in the relative proportion of sugars were an increase in galactose from 2% to 41% and a decrease in sucrose and glucose from 30% to 2% and from 55% to 45%, respectively. It is generally understood that the rapid loss of saccharides happens due to them being among the first to be consumed by micro-organisms [44,45]. Leaching should, however, be considered as a possibility, especially as a consequence of the steam released during pile storage [5,28,46]. Concentrations of galactose and mannose in the middle of the pile by leaching might have been observed here. In his dissertation, Sauro Bianchi noted the prevalence of hemicellulose-derived saccharides in water extracts above 80 °C [46]. Noting that the extraction temperature that was used in this study was 120 °C, the presence of saccharides from hemicellulose should be expected. The presence of mannose after storage for 4 weeks and the increased amount of galactose may be, at least partly, explained by the degradation of galactoglucomannan, the main water-soluble hemicellulose in Norway spruce [47]. As a polymeric carbohydrate, galactoglucomannan would be included in the ‘unidentified’ hydrophilic extractive group (Figure 4). It is also worth noting that the degradation of lactose (4-*O*-β-d-galactopyranosyl-d-glucopyranose) released galactose units. 

#### 2.3.7. Sugar Alcohols

The quantified amount of sugar alcohols in the hydrophilic bark extracts determined using GC-FID/MS is presented in Figure 10. A significant overall variation was observed in the amount of sugar alcohols during storage. After storage for 4 weeks, a sharp increase was detected in the sugar alcohol concentration in the middle of the pile, whereas on the side and at the top of the pile, the total amount remained the same. After 4 weeks, maltotriitol and isomaltitol almost disappeared, whereas inositol and maltitol dramatically increased. Moreover, l-ribulose and erythritol were produced. At the end of the 24-week storage, the amount of sugar alcohols significantly decreased, with only the middle of the pile having a slightly elevated amount of total sugar alcohols. The most significant changes in the relative amount of individual sugar alcohols in the samples (zero sample vs. 24-week storage) were an increase in arabitol, mannitol and l-ribulose from 5% to 23%, from 4% to 16% and from 1% to 11%, respectively, and a decrease in pinitol and maltotriitol from 62% to 29% and from 12% to 0%, respectively. The literature regarding the storage of wood and forestry sidestreams does not discuss the fate of sugar alcohols much. Our previous study regarding the storage of sawlog bark found the sugar alcohol levels to remain constant (c.a. 10 mg/g level) during winter storage until week 12 and then drop to 3 mg/g at 24 weeks of storage [10]. The increase in sugar alcohols observed here, at week 4, should probably be attributed to the hydrogenation reactions of sugars—a process that has also been utilised in the production of value-added chemicals and food ingredients [48]. It is possible that the initial conversion of some sugars to sugar alcohols happened followed by their rapid leaching towards the middle of the pile. This would include the conversion of maltose to maltitol. Production of l-ribulose would, however, suggest a microbial and enzymatic conversion [49]. Similarly the formation of inositol happens through enzymatic phosphorylation of glucose to glucose phosphate (see the residues in Figure 9) followed by isomerisation of glucose phosphate to inositol-phosphate and finally dephosphorylation to inositol [50].

#### 2.3.8. Organic Acids

The quantified amount of organic acids in the hydrophilic bark extracts determined using GC-FID/MS is presented in Figure 11. A considerable overall decrease was observed in the amount of organic acids during storage. At the beginning of storage, gluconic acid, citric acid and quinic acid constituted the vast majority of all organic acids. The presence and leaching of organic acids during wood storage has been noted several times before [28,51]. According to Fuller, the presence of even mild acetic acid in pile storage can lead to the shortening of the cellulose fragments in wood [5]. The most significant changes in the relative proportion of organic acids in the samples (zero sample vs. 24-week sample) were an increase in l-glutamic acid from 1% to 43% and a decrease in citric acid and quinic acid from 28% to 4% and from 30% to 10%, respectively. Notably, the concentration of organic acids on the side and at the top of the pile decreased rapidly, whereas in the middle of the pile, an increase was observed from week 12 to week 24. Contrary to these results, the production of new organic acids was not observed in our previous study regarding sawlog storage of bark [10]. Generally, l-glutamic acid is an amino acid by-product of microbiological fermentation of plant proteins (e.g., gluten) with, for instance, glucose as the carbon source [52]. Thus, the significant increase observed in l-glutamic acid also indicated an increase in microbial degradation during storage. Among other degradation products, 2,3-dihydroxysuccinic acid (tartaric acid) was also formed as a fermentation product—a common degradation product in aged fruits and wines [53]. 

#### 2.3.9. Stilbenes

Stilbenes are among the most attractive organic compounds and potential platform chemicals obtained from spruce bark. However, stilbenes are usually lost at a particularly fast rate, not only because they are hydrophilic and may be leached by rainwater but also because of their high anti-oxidative capacity and reactivity under UV light to form phenanthrene derivatives via photo-oxidative cyclisation [18].

The quantified amount of stilbenoids in the hydrophilic bark extracts determined using GC-FID/MS is presented in Figure 12. During storage, a radical overall loss of stilbenes was observed in the study samples, especially during the first few weeks of storage. After storage for 4 weeks, only 23% of the original stilbenes remained, and the stilbene monoglucosides isorhapontin, astringin and piceid, totalling 90% of the original monoglucosides, were almost completely removed. However, the concentrations of the aglycones resveratrol, piceatannol and rhapontigenin increased by 23% at week 4 as a result of the hydrolysis reactions of the glucosides. Moreover, distilbenes and sesquistilbenes constituted 63% of the total stilbenes at the beginning of storage, but only 13% and 6% of the original distilbenes and sesquistilbenes, respectively, remained at the end of storage.

The average concentrations of stilbene monoglucosides, sesquistilbenes and distilbenes in piled bark (from both covered and non-covered piles) were found to be 21.2, 18.7 and 15.8 mg/g of dry matter, respectively. On the other hand, as reported in a previous study, the average amounts of stilbene monoglucosides, sesquistilbenes and distilbenes in the bark of freshly felled (winter-stored) saw logs were found to be 23.5, 10.8 and 10.1 mg/g of dry matter, respectively [10]. Thus, it seems that while the initial amount of stilbene monoglucosides in bark pile and sawlog bark is closely paralleled, the amount of sesqui- and distilbenoids is greater in chipped and piled bark. This may be coincidental, given that the stilbene levels of individual saw logs may considerably vary. However, while the initial amount of stilbenoids was slightly greater in the piled bark, after just 4 weeks of storage, the winter-stored saw logs retained 79% more stilbenoids than those retained by the piled bark. This finding highlights the impact that the storage method can have on individual extractives. To effectively utilise piled bark for its stilbene content, either protective measures need to be taken to ensure their preservation, or the bark needs to be further processed rapidly (within days of the initial piling). 

Stilbene concentrations presented here were markedly higher than those reported by Krogell et al. [21]; however, Jyske et al. have reported at least twice as high concentrations of stilbene glucosides in the bark of 18–37 year old *Picea abies* trees [54]. It should, however, be noted that while Jyske et al. [54] looked at stilbene concentration at different bark zones and heights (inner bark having highest stilbene concentrations), our results reflect more the average stilbene concentration in sidestream *Picea abies* bark from sawmills without further distinctions. Stilbene levels similar to those presented by Jyske et al. [54] have also been reported in the root bark of Norway spruce [55].

#### 2.3.10. Flavonoids

The quantified amount of simple flavonoids in the hydrophilic bark extracts determined using GC-FID/MS is presented in Figure 13. The initial amount of flavonoids was approximately twice as high as that reported by Krogell et al. [21].The loss of flavonoids seemed to follow a path similar to that of stilbenes, with a dramatic concentration decrease after just 4 weeks of storage. Slower flavonoid degradation was observed in our previous study regarding *Picea abies* sawlog bark [10]. The most prominent flavonoids were taxifolin glycoside, naringin, catechin, taxifolin and neohesperidin. Notably, dihydromyricetin, which has potent anti-oxidative properties, was found to be the most resilient among flavonoids [56]. Its amount was even found to be somewhat increased during storage (e.g., through the bio-conversion of other flavonoids). The most significant changes in the relative proportion of extractives in the samples (zero sample vs. 24-week sample) were an increase in dihydromyricetin and naringenin chalcone from 5% to 59% and from 5% to 23%, respectively, and a decrease in taxifolin glycoside, naringin, catechin and neohesperidin from 23%, 23%, 17% and 11% to 0%, respectively. Flavonoids (similarly to stilbenes) are lost particularly rapidly to photo-degradation because of their tendency as phenolic compounds to form unstable phenoxy radicals [12,13,18].

### 2.4. High-Performance Liquid Chromatography (HPLC) Analysis of Proanthocyanidins

**Figure 14:**
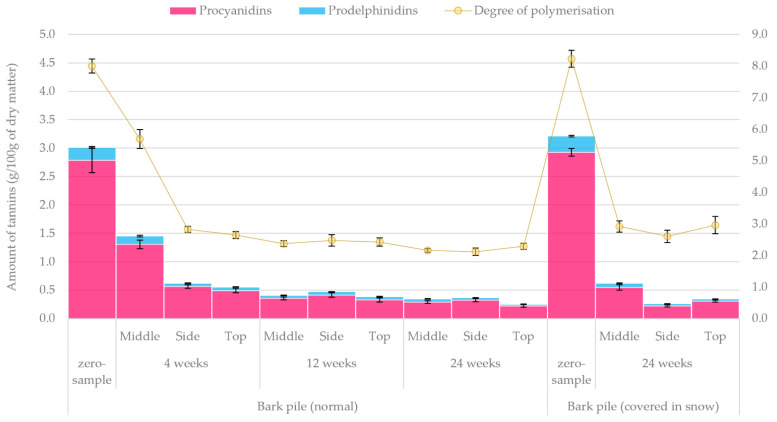
Quantified amounts of tannins (procyanidins, prodelphinidins) and the degree of polymerisation (DP) in freeze-dried bark samples under pile storage.

Overall, the thiolytic degradation of spruce bark CTs (procyanidins) produced (epi)catechins and (epi)catechin thioethers as major reaction products and (epi)gallocatechins and (epi)gallocatechin thioethers as minor products, indicating that spruce bark CTs are a mixture of procyanidins and prodelphinidins, as observed in previous studies [11,57,58,59]. As shown in Figure 14, the initial CT content was 3.0–3.2 g/100 g, but it decreased rapidly during storage. After 4 weeks, the total content of CTs was found to exhibit a great variation (0.556–1.451 g/100 g) between the different samples, but this variation always remained below 50% of the original amount. After 12 weeks, the concentration reached 0.384–0.472 g/100 g, and only minor changes were observed for the rest of the storage duration. The final CT content in the normally stored bark pile was found to be 0.251–0.365 g/100 g after 24 weeks, which is equal to approximately 10% of the original content. A recent study on Scots pine reported a similar drastic and rapid loss in the CT content during pile storage of bark [23].

The average CT content in the snow-covered piles was somewhat higher after storage for 24 weeks, and notable differences were observed between the samples. These samples were obtained from different pile locations, which might partly explain the variations observed in the CT content. In this study, the highest CT content was determined twice in the samples taken from the middle of the pile (after storage for 4 weeks and 24 weeks for normally stored and snow-covered piles, respectively). It is possible that the bark in the middle of the pile was better protected from environmental stress than that on the side or at the top of the pile. This also means that the CTs were less exposed to detrimental reactions. Similarly, a recent study has shown that the outer bark is expected to protect the inner bark, with the CTs in the outer bark degrading much faster than in the inner bark during the summertime fresh-air storage of spruce logs [11]. However, further research is still needed to confirm the significance of location in a pile for the recovery of CTs and other constituents in spruce bark.

The average DP in spruce bark CTs was found to be the highest at the beginning of the experiment, but it decreased during storage, indicating that the polymerisation of CTs is the first step in the degradation process. However, the oxidation of CTs during storage might result in degradation and the formation of new covalent bonds between CTs and other macromolecules, producing new polymers partially resistant to thiolysis [59,60]. As a result, both the content and the DP of CTs are somewhat under-estimated with the current determination method. Furthermore, the relative proportion of prodelphinidins in CTs was found to slightly increase during storage. The same finding was observed in the CTs of spruce logs stored in the open air [11]. This may indicate that prodelphinidins in spruce bark CTs are more resistant to environmental stress than procyanidins.

### 2.5. Carbohydrate Analysis

#### 2.5.1. Acid Hydrolysis and High-Performance Anion-Exchange Chromatography (HPAEC) Analysis of Monosaccharides

**Figure 15:**
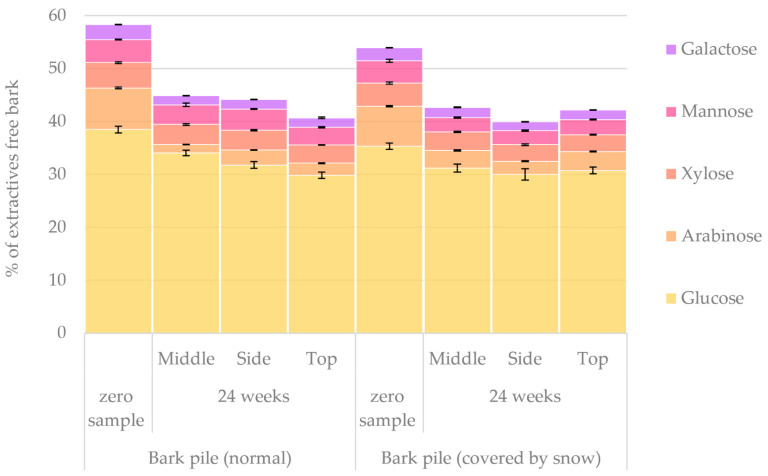
Quantified amounts of holocellulosic monosaccharides in bark samples at the beginning and end of normal and snow-covered pile storage.

The results obtained from the HPAEC analysis of extractive-free bark monosaccharides (i.e., holocellulose) are presented in Figure 15. The initial amount of holocellulosic monosaccharides in the bark samples was found to be 58% in extractives-free bark in the normal bark pile and 54% in the snow-covered pile. After a storage period of 24 weeks, the amount in both piles decreased to approximately 42% of extractives-free bark. These values, however, correlate to approximately 35.8% of the initial amount of holocellulose in dry bark (according to Figure 1) and 37.5% in dry bark after 24 weeks of storage. Thus, the total holocellulose content (as % of dry bark) increased 1.7%. In our previous study regarding *Picea abies* sawlog bark storage, the amount of holocellulose was initially 33.9% of dry bark and increased to 37.7% in 24 weeks (a 3.8% increase) [10]. Čabalova et al. also reported relatively increased cellulose content during storage for 8 months [25]. Generally, glucose was by far the most prominent monosaccharide. The notable changes in the relative proportion of monosaccharides (zero sample vs. 24-week sample) were an increase in glucose from 66% to 74% of dry matter and a decrease in arabinose from 13% to 5% of dry matter. Moreover, mannose decreased slightly more in the samples from the snow-covered pile. 

#### 2.5.2. Acidic Methanolysis

**Figure 16:**
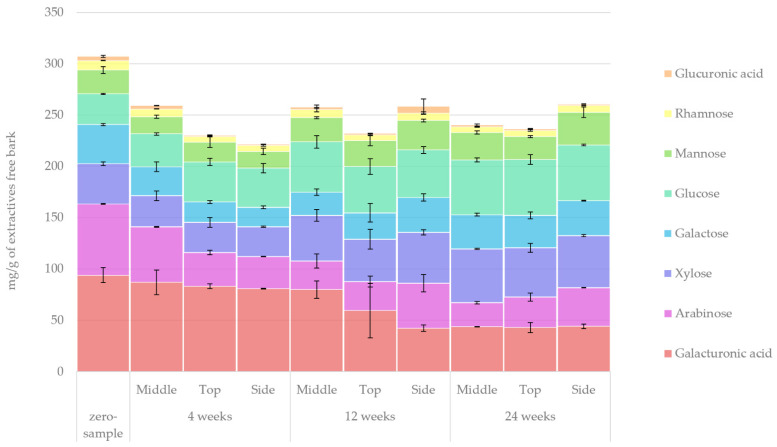
Quantified amounts of hemicellulosic carbohydrates in the extractive-free bark samples under normal pile storage.

The results obtained from the acidic methanolysis of extractive-free bark monosaccharides (i.e., hemicelluloses) are presented in Figure 16. These results indicate that the overall amount of hemicelluloses decreased by 21%. Such a decrease occurred during the first 4 weeks of storage, and the total amount of hemicellulosic monosaccharides remained constant throughout the storage period, although changes in the composition occurred. The most notable changes in the relative proportion of hemicellulosic groups in the samples (zero sample vs. 24-week sample) were an increase in glucose and xylose from 10% to 22% and from 13% to 21%, respectively, and a decrease in galacturonic acid and arabinose from 31% to 18% and from 23% to 12%, respectively. Conversion of galacturonic acid to galactose was probably also observed. A similar trend was observed with regard to the sampling location in each pile, and the concentration of extractives was probably also observed at weeks 4 and 12. The highest concentration was found in the middle of the piles, whereas the top and side of the piles showed greater signs of degradation. Notably, the hemicellulosic monosaccharides presented here are basically a subset of the results presented in Figure 15. By comparing the results for holocellulosic and hemicellulosic monosaccharides (in the normal bark pile), we were able to observe that the cellulosic monosaccharides were primarily composed of glucose and mannose. The apparent increase in some hemicelluloses, such as glucose and xylose, could be explained (similarly to the increase in lignin in bark (see Section 2.1.2)) as a relative increase caused by the faster degradation of extractives and other carbohydrates. Relative increases in hemicelluloses were also observed in our previous study regarding single stem *Picea abies* bark storage [10]. It should also be noted that while the total amount of carbohydrates as mg/g of extractives-free bark (in Figure 15) decreased, the relative amount of carbohydrates as % of dry bark (i.e., bark containing extractives; see Figure 1) slightly increased during storage. A similar relative increase in cellulose and lignin due to short term storage of *Picea abies* bark has also been recently reported by Čabalova et al. [25]. This effect could be likened to the concentration of carbohydrates by weight observed in dried fruits.

## 3. Materials and Methods

### 3.1. Experimental Setup of Storage Studies and Sampling

**Figure 17:**
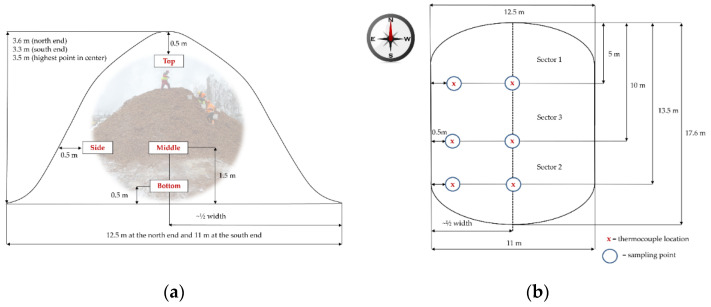
Schematic representations of the measures of the bark pile and (**a**) the thermocouple locations inside the bark pile and (**b**) the sampling points and sectors.

All the bark used in this study was provided by the UPM-Kymmene Oyj sawmill in Ostrobothnia, and all the bark pile setups were located outside in the factory yard in Pietarsaari. The two 450 m^3^ bark piles used in this storage study were constructed on 20 and 21 February 2017. The piles consisted of *Picea abies* bark that was debarked a maximum of 48 h before the construction of the pile. However, most of the material was even fresher. It should be noted that since the bark originated as a sidestream from a standard operating sawmill, no exact measurements of individual trees from which the bark was obtained (their height, width, age, etc.) were available. It is known that the used trees were gathered within a 200 km range from Pietarsaari, mostly from private forest owners in Ostrobothnia. The sampling points and dimensions of the bark in the non-covered pile are outlined in Figure 17. The sampling locations were chosen from areas of piles expected to have significant variations in temperature and moisture content, according to earlier storage studies [29]. The length of the constructed pile was 17.6 m, and it was divided into three sectors. Sector one was opened for sampling after 4 weeks, sector two after 12 weeks and finally sector three at the end of the storage study, after 24 weeks. Thermocouples were placed inside the pile in the locations indicated in Figure 17a, and the temperature was measured in each sector until the sector was opened for sampling. At each sampling time, bark samples were taken from the exact locations of the thermocouples, except for the bottom of the pile.

### 3.2. Sample Pre-Treatment and Basic Characterisation

First, the bark was ground to a finer particle size with a Jens Algol System woodchipper (Jenz GmbH, Petershagen, Germany). Then, a standard method (CEN/TS 14774-2:2004) was used to determine the fresh bark samples’ moisture content [61]. Next, the samples were dried at 105 °C until a constant mass was achieved. All measurements were performed in duplicate.

The bark was then lyophilised (for at least 3 days) and ground with a Retsch SM 100 cutting laboratory mill (Retsch GmbH, Haan, Germany) equipped with a bottom sieve with trapezoidal holes (perforation size < 1.0 mm) for chemical analysis. Samples were stored in a frozen state (below −20 °C). Then, the dry matter content of the lyophilised bark samples was determined by drying 1 g of bark powder at 105 °C in an oven overnight in tared crucibles. 

### 3.3. Calorific Values and Carbon, Hydrogen and Nitrogen (CHN) Measurements of Bark Samples

First, the moisture content (on a wet basis) of the bark samples was analysed according to the same method as referred to in Section 3.2, and the ash content was determined according to the standard method SFS-EN 14775 [62]. A bomb calorimeter (IKA C 5000; IKA-Werke GmbH & Co., Staufen, Germany) was used to determine the calorific heating value (qp_gross_) of the bark dry matter. Samples were dried, milled (Retsch SM-1 mill; Retsch GmbH, Haan, Germany) and pelletised before analysis with the bomb calorimeter. Next, the calorimetric heating values were determined and the gross calorific values were calculated using the standard method CEN/TS 14918:2005 [63]. Then, the carbon, hydrogen and nitrogen concentrations were analysed using the standard method SFS-EN ISO 16948:2015 at the laboratory of Ahma Environment Ltd. [64]. The following equation was used to calculate the effective heating value (qp_net_):

qp_net_ = qp_gross_ − 2.45 × 0.09H_2_ (1)

where qp_net_ is the effective heating value (kJ·kg^−1^), qp_gross_ is the calorific heating value (kJ·kg^−1^), 2.45 MJ kg^−1^ is the latent heat of vaporisation of water at 20 °C, 0.09 is a factor expressing that one part of hydrogen and eight parts of oxygen form nine parts of water, and H_2_ is the hydrogen content of the oven-dried biomass.

### 3.4. Chemicals

The solvents used in the sample preparation of extractives were analytical-grade acetone (BDH), HPLC-grade *n*-hexane (VWR), methyl *tert*-butyl ether (Lab-Scan), pyridine (BDH), 95% ethanol (EtOH, >94%, ETAX A; Altia Corporation) and *n*-butanol (Merck). Bis(trimethylsilyl)trifluoroacetamide (BSTFA) and trimethylchlorosilane (TMCS) were obtained from Regis Technologies (Morton Grove, IL, USA) for silylation.

The compounds used as internal standards in the GC analysis of extractives were heneicosanoic acid (99%; Sigma-Aldrich Finland, Espoo, Finland) and betulinol (≥98%; Sigma-Aldrich Finland, Espoo, Finland), cholesteryl margarate (≥97%; TCI, Portland, OR, USA) and 1,3-dipalmitoyl-2-oleoylglycerol (≥99%; Sigma). NaOH (>98%; VWR), HCl (37%; VWR), Na_2_CO_3_ (≥99.8; Sigma-Aldrich Finland, Espoo, Finland), sulphuric acid (95–97%; Sigma-Aldrich Finland, Espoo, Finland), and bromocresol green (>95%; Sigma-Aldrich Finland, Espoo, Finland) were also used in the analysis.

Cysteamine (≥98%; Sigma-Aldrich Finland, Espoo, Finland), 37% aqueous hydrochloride (Thermo Fisher Scientific, Waltham, MA, USA) and HPLC-grade methanol (≥99.8%; VWR International, Helsinki, Finland) were used for the thiolysis of CTs. Then, CT degradation products, that is, free flavan-3-ols (terminal units) and their cysteaminyl derivatives (extension units), were quantified using external standards of catechin, epicatechin, gallocatechin and epigallocatechin (Sigma-Aldrich Finland) and thiolysed procyanidin B2 (Extrasynthese, Lyon, France). HPLC-grade acetonitrile (VWR International) and formic acid (≥98%; Sigma-Aldrich, Espoo, Finland) were used for HPLC determination of thiolysed CTs.

### 3.5. ASE Extraction 

Bark samples were extracted with a Dionex Accelerated Solvent Extractor (Dionex, ASE 100, Sunnyvale, CA, USA) using *n*-hexane and water as solvents to extract lipophilic and hydrophilic extractives, respectively. The extraction temperature was set to 120 °C, with a static extraction time of 10 min, flush of extraction cell of 60%, nitrogen purge for 70 s and extraction pressure of 1500 psi. For each extraction procedure, 2 g of bark powder was loaded to a 34 mL extraction cell plugged with a cellulose filter. Each sample was first extracted with *n*-hexane and then with water, and the extractive-free bark was consequently lyophilised and stored for carbohydrate analysis. All extraction procedures were performed in duplicate for each sample.

### 3.6. Gravimetric Analysis of Total Dissolved Solids and Preparation of Stock Solutions

Overall, the TDSs of bark extracts were determined gravimetrically. The *n*-hexane extracts were evaporated to near dryness in a rotary evaporator and subsequently transferred to tared Kimax test tubes in acetone and finally evaporated to dryness under nitrogen flow. The weight of the dried extract was the TDS of the *n*-hexane extracts. A stock solution (100 mL) was then prepared by dissolving the extract in acetone.

Stock solutions of hydrophilic extracts were prepared by diluting the raw extract to 100 mL with ultra-high-quality (UHQ) water. Some of the stock solutions (10 mL) were lyophilised, and the TDS of the hydrophilic extracts was determined according to the weight of the lyophilised sample. 

### 3.7. Analysis of Bark Extractives with Chromatographic Methods 

#### 3.7.1. Qualitative Analysis of Bark Extracts by Gas Chromatography with Mass Selective Detection (GC-MS)

To perform a qualitative analysis, 3 mg of extracts (based on dry weight) was dried (either by nitrogen flow or lyophilisation) and dissolved in 500 μL of pyridine and 300 μL of a silylation reagent (TMCS). The silylation process was accelerated by keeping the sample in a 70 °C oven for 1 h. The samples were then analysed using a Hewlett Packard 5973 GC-MS instrument equipped with an HP-5 column (30 m × 0.32 mm, with a 0.25 μm film). Next, the samples were injected at 290 °C and detected with a mass selective detector at 300 °C. Notably, the method used for the analysis was the same as in our previous study [10]. 

#### 3.7.2. Quantitative Analysis of Bark Extracts by GC-FID

To perform a quantitative analysis, approximately 3 mg samples of bark extracts were dried with internal standards. The mixtures were then dissolved in 500 μL of pyridine and 300 μL of a silylation reagent and kept in an oven for 1 h. 

To analyse the extractive groups, 100 μg of four internal standards was used: heneicosanoic acid, betulin, cholesteryl margarate and 1,3-dipalmitoyl-2-oleoylglycerol. An Agilent 6850 GC-FID instrument equipped with a short HP-1/simulated distillation column (7.5 m × 0.53 mm, with a 0.15 μm film) was used for the analysis. The samples were injected on-column at 90 °C and detected using FID at 320 °C. The temperature program used was the same as in our previous study [10]. 

To perform an individual extractive analysis, 100 μg of heneicosanoic acid and the same amount of betulin were added as internal standards. An Agilent 6850 GC-FID instrument equipped with a long HP-5 column (30 m × 0.32 mm, with a 0.25 μm film) was used for the analysis. The samples were then injected at 290 °C and detected at 300 °C. The temperature program used was the same as in our previous study [10].

To analyse the esterified lipophilic extractives, the samples were hydrolysed and derivatised for analysis as described by Halmemies et al. (2021) [10].

#### 3.7.3. Analysis of Proanthocyanidins by High-Performance Liquid Chromatography (HPLC)

A thiolytic degradation method as described by Korkalo et al. was applied to determine CTs (proanthocyanidins) in the lyophilised bark samples [65]. First, a ground sample (10–20 mg) was mixed with 1 mL of a depolymerisation reagent (3 g of cysteamine dissolved in 56 mL of methanol acidified with 4 mL of 13 M HCl) and incubated for 60 min at 65 °C. During incubation, the samples were vortexed for a few seconds every 15 min. Thiolysis was stopped by transferring the samples into an ice bath. The cooled samples were then filtrated into HPLC vials and analysed on an Agilent 1290 Infinity UHPLC instrument equipped with a Zorbax Eclipse Plus C_18_ column (50 × 2.1 mm i.d., 1.8 m; Agilent Technologies, Santa Clara, CA, USA). The binary mobile phase consisted of 0.5% formic acid (aq.) and acetonitrile. Elution was started with 2% acetonitrile isocratically for 2 min, followed by a linear gradient to 5% in 3 min, to 15% in 7 min, to 20% in 3 min, to 35% in 5 min, to 90% in 1 min and back to the initial condition in 2 min. The post-time was 2 min before the next injection. The flow rate was 0.5 mL/min, and the injection volume was 2 µL. Elution was monitored using diode array detection (DAD; λ_1_ = 270 nm, λ_2_ = 280 nm) and fluorescence detection (FLD; λ_ex_ = 275 nm, λ_em_ = 324 nm). 

### 3.8. Carbohydrate Analyses

Acid hydrolysis and acidic methanolysis were used to analyse the carbohydrate (holocellulose, cellulose and hemicelluloses) and acid-soluble and acid-insoluble lignin (see below) content of extractive-free bark. Holocellulose is defined as the sum of cellulosic and hemicellulosic carbohydrates. The holocellulose and lignin content was first determined using acid hydrolysis, and then the hemicellulose content was determined using acidic methanolysis. Then, the cellulose content of the samples was determined as the difference between holocellulose and hemicelluloses.

#### 3.8.1. Acid Hydrolysis

Separation of holocellulose, acid-insoluble lignin and acid-soluble lignin from the extractive-free bark samples was performed according to the TAPPI standard T 222 [66]. For the acid hydrolysis samples, 200 mg of lyophilised extractive-free bark was weighed in a test tube. Then, around 4 mL of 72% cold sulphuric acid was added, and the test tubes were kept in a water bath at 30 °C for 1 h. Every 5 min, the mixtures were stirred with a glass rod. Next, the samples were transferred to 250 mL autoclave bottles, washed with 112 mL of UHQ water and then placed in an autoclave (MELAG Autoklav 23, Berlin, Germany) at a pressure of 1 bar (~121 °C) for 1 h. 

Solid acid-insoluble lignin was then separated from the mixtures by filtration with a tared borosilicate glass filter (Munktell MGA 413004, Falun, Sweden) in a vacuum funnel. Insoluble lignin was gravimetrically determined by drying the residues together with the used filter papers (of known weight) in an oven at 105 °C to a constant weight. The filtrates were then diluted to 500 mL with UHQ water and consequently analysed with HPAEC for their holocellulose-derived monosaccharide content and with UV–Vis spectroscopy for their soluble lignin content. 

#### 3.8.2. High-Performance Anion-Exchange Chromatography (HPAEC) Analysis of Holocellulose-Derived Monosaccharides

First, HPAEC was used to analyse the monosaccharides formed during the acid hydrolysis from the 500 mL dilutions. Standard solutions for HPAEC were prepared using a sulphuric acid concentration corresponding to the samples’ background: cold 72% sulphuric acid (3 mL) was diluted to 500 mL with UHQ water. Fucose (500 ppm) was used as an internal standard. The preparation of the standard solutions is described in detail in our previous study [10]. 

Bark samples (500 mL UHQ water dilution) from acid hydrolysis were analysed with HPAEC (Dionex) using 1 M sodium acetate, 0.5 M sodium acetate plus 0.1 M NaOH and 0.3 M NaOH solutions as eluents. The analytes were then separated in CarboPac PA1 + Quard PA1 columns and detected with an ED50 detector using carbohydrate pulsing. The post-column elute was pumped by an IC25 isocratic pump.

Samples for HPAEC analysis were prepared by pipetting 2 mL of an internal standard solution to a 20 mL volumetric flask and filling the flask with the diluted sample (500 mL) from the acid hydrolysis. This solution (1.0–1.5 mL) was then transferred into an HPLC vial by filtrating it through a syringe filter (Phenex-RC, 0.2 μm).

#### 3.8.3. UV–Vis Measurement of Acid-Soluble Lignin

The amount of acid-soluble lignin was determined from the 500 mL dilution following acid hydrolysis via UV–Vis spectroscopy at 205 nm according to the TAPPI standard UM 250 using an extinction coefficient of 120 L/(g·cm) (for softwood) [67].

#### 3.8.4. Acidic Methanolysis

The amount of hemicellulose in spruce bark samples was analysed from extractive-free lyophilised bark using acidic methanolysis. An internal standard solution was prepared by dissolving 10 mg of sorbitol into 100 mL of methanol. To prepare an external standard solution, a 10 mg mixture of arabinose, galactose, glucose, xylose, mannose, galacturonic acid and glucuronic acid was dissolved into 100 mL of UHQ water. Then, a methanolysis reagent was prepared by cooling 100 mL of methanol in an ice bath and carefully adding and mixing 16 mL of acetyl chloride into the cold methanol. Next, the reagent was stored at −20 °C.

For methanolysis, 2 mL of the methanolysis reagent was added to 2–3 mg of extractive-free bark samples and to a dried monosaccharide standard sample (1 mL). The samples were then sonicated in an ultrasound bath and kept at 100 °C in an oven for 3 h. Pyridine (80 μL) and an internal standard (1 mL) were next added to the samples, and the solvent was evaporated. Then, 80 μL of pyridine and 250 μL of a silylation reagent were added, and the samples were sonicated in an ultrasound bath and kept in a shaker at room temperature for 40 min. Next, the samples were filtrated with glass wool for GC analysis with an Agilent 6850 gas chromatograph equipped with an HP-5 column (30 m × 0.32 mm, with a 0.25 μm film). Finally, the samples were injected at 260 °C and detected by FID at 290 °C. The method used was the same as in our previous study [10].

### 3.9. Statistical Analysis

One-way analysis of variance (ANOVA) was used to assess the effect of storage time (0, 4, 12 or 24 weeks) and sampling location (side, middle or top of the pile) on the concentrations of bark components, DP values of CTs, ash content and effective heating value. Logarithmic transformation was used for the variables that were sufficiently non-normal to cause concern about the validity of the normality assumption. In addition, the Kruskal–Wallis test, the non-parametric equivalent of one-way ANOVA, was used for the variables that were not normally distributed even after logarithmic transformation.

In turn, an independent-samples *t*-test was used to test the statistical differences in the concentrations, DP, ash content and net calorific value between the snow-covered and non-covered bark piles. Again, logarithmic transformation was used for the non-normal variables.

## 4. Conclusions

According to our study of bark storage in piles (both non-covered and covered with snow), spruce bark is a valuable raw material that is rich in hydrophilic extractives. However, it is worth noting that the material losses experienced as a result of pile storage (even during the winter) are dramatic, even after only a few weeks of storage. The loss of hydrophilic, phenolic extractive groups, such as stilbenes and tannins, was particularly notable. Significant proportions of the losses of extractives are to be attributed to the microbiological degradation and increase in pile temperature, which initiates and facilitates further degrading chemical reactions. In addition, exposure to UV light and the leaching of extractives from piles also cause losses of these compounds.

A clear trend was observed with regard to the sampling location in storage piles and the concentrations of the studied chemical compounds. In particular, both extractives and carbohydrates were found to have high concentrations in the middle of the pile with prolonged storage durations, indicating in some instances a leaching of compounds from elsewhere in the piles. Despite the covering of the other pile, no significant difference was observed between the degradation results of the non-covered and snow-covered bark piles. Other covering options and their impact on bark extractives should be further investigated.

From the results, it was evident that if piled bark material is to be valorised for its extractive content, storage periods should be as short as possible. Even four weeks of piled bark storage seems too long a time for stilbenes and tannin products. It was demonstrated that a simple hydrophilic extraction (e.g., with hot water) can effectively remove many potential platform chemicals for further purification steps. Methods, such as these, ought to be considered especially by bio-refinery plants that handle sidestream bark material. In addition, the logistics of bark material delivery to refineries needs to be planned in order to eliminate unnecessary exposure to weathering and moisture.

## Figures and Tables

**Table 1:** Results (*p*-values) obtained from testing the statistical differences among the storage duration (0, 4, 12 or 24 weeks), sampling location (middle, side or top) and snow cover (covered or not covered with snow) in terms of the amounts of lipophilic extractives, hydrophilic extractives, condensed tannins (CTs) and total dissolved solids (TDSs). The bold text indicates a statistically significant difference with a *p*-value less than 0.10.

	Storage Time	Sampling Location	Snow Cover
Lipophilic Extractive Groups			
Resin acids	0.280	0.148	**0.018**
Fatty acids	0.313	0.115	0.285
Diterpenoids	**0.058**	0.651	0.157
Sterols	0.236	0.431	0.464
Other lipophilic extractives	0.379	0.166	0.157
Unidentified	**0.022**	0.142	**0.005**
Steryl esters	**0.0** **66**	0.446	0.255
Triglycerides	**<0.001**	0.764	0.200
Hydrophilic Extractive Groups			
Sugars	0.355	**0.0** **78**	0.344
Organic acids	0.527	**0.0** **10**	0.400
Sugar alcohols	0.219	0.192	0.432
Stilbenes	**0.039**	0.670	0.170
Flavonoids	**0.0** **23**	0.430	0.176
Other phenolics	**0.0** **31**	0.404	0.458
Alcohols	**0.076**	0.233	0.319
Lignans	0.124	0.133	0.234
Other hydrophilic extractives	0.795	**0.0** **68**	0.472
Sesquistilbenes	**0.0** **02**	0.862	**n/a**
Distilbenes	**<0.001**	0.805	**n/a**
Unidentified	**0.0** **05**	0.719	0.499
Condensed Tannins			
Total concentration	**0.039**	0.733	0.827
Procyanidins	**0.039**	0.733	0.827
Prodelphinidins	**0.0** **25**	0.424	0.436
DP	**0.039**	1.000	**0.0** **05**
TDSs			
*n*-Hexane extract	0.288	0.201	0.324
Hot-water extract	**0.006**	0.161	0.364
Biofuel Properties of Stored Bark			
Ash content	0.117	0.233	0.103
Effective heating value	0.280	0.153	**0.024**

**Table 2:** Moisture, ash, carbon, hydrogen and nitrogen content of the studied bark samples and their effective heating values.

Storage Time, Weeks	Sampling Location	Moisture Content, %	Ash Content, %	Carbon Content ^1^, %	Hydrogen Content ^2^, %	Nitrogen Content ^3^, %	Effective Heating Value, MJ/kg
Normal Pile							
0		57.38 ± 0.68	3.21 ± 0.02	51.4	5.82	0.47	19.14 ± 0.02
4	Middle	59.89 ± 1.05	3.30 ± 0.01	51.3	5.80	0.53	19.10 ± 0.01
4	Side	52.20 ± 1.22	3.53 ± 0.01	52.2	5.74	0.52	19.40 ± 0.01
4	Top	56.92 ± 0.64	3.46 ± 0.02	52.1	5.78	0.53	19.56 ± 0.03
12	Middle	61.40 ± 0.86	3.45 ± 0.01	51.1	5.73	0.53	18.78 ± 0.00
12	Side	53.09 ± 0.81	3.75 ± 0.02	51.7	5.63	0.55	19.37 ± 0.01
12	Top	51.65 ± 0.32	3.74 ± 0.01	52.2	5.59	0.54	19.40 ± 0.02
24	Middle	57.83 ± 0.40	3.53 ± 0.05	52.5	5.71	0.52	19.48 ± 0.01
24	Side	40.79 ± 0.82	3.85 ± 0.00	52.5	5.50	0.56	19.47 ± 0.02
24	Top	61.01 ± 0.71	4.17 ± 0.04	52.8	5.45	0.60	19.52 ± 0.01
Snow-Covered Pile							
0		56.01 ± 0.89	3.12 ± 0.01	51.3	5.77	0.47	19.11 ± 0.01
24	Middle	62.05 ± 0.73	3.77 ± 0.12	51.8	5.65	0.50	19.36 ± 0.01
24	Side	64.33 ± 0.44	4.92 ± 0.08	51.5	5.34	0.61	19.09 ± 0.02
24	Top	69.50 ± 0.45	8.47 ± 0.35	49.9	5.27	0.56	18.13 ± 0.02

^1^ Measurement uncertainty ±2%. ^2^ Measurement uncertainty ±4%. ^3^ Measurement uncertainty for values <0.3 is ±30%, and for values >0.3 is ± 15%.

## Data Availability

Detailed data regarding the presented figures are available within the Supplementary Materials. Other data are available upon request from the corresponding author.

## Supplementary Materials

The following supporting information can be downloaded online, Table S1: Values for Figure 1, Table S2: Values for Figure 3, Table S3: Values for Figure 4, Table S4: Values for Figure 5, Table S5: Values for Figure 6, Table S6: Values for Figure 7, Table S7: Values for Figure 8, Table S8: Values for Figure 9, Table S9: Values for Figure 10, Table S10: Values for Figure 11, Table S11: Values for Figure 12, Table S12: Values for Figure 13, Table S13: Values for Figure 14, Table S14: Values for Figure 15, Table S15: Values for Figure 16.

## Sample Availability

Samples discussed in this article are not available for further study.
