# Supplementary material for: Behaviour of Extractives in Norway Spruce (Picea abies) Bark during Pile Storage
Source: Molecules. 2022 Feb 10;27(4):1186. doi: 10.3390/molecules27041186 (PMC8878638; doi:10.3390/molecules27041186)
Supplement: Supplementary file 1 [file molecules-27-01186-s001.zip › molecules-1568707-supplementary.pdf]

molecules

MDPI

Supporting material

Tabulated values of the results presented in the article.

Table S1. Values for Figure 1.

**Bark Pile**	**UC** [a]	**UC**	**UC**	**UC**	**UC**	**UC**	**UC**	**UC**	**UC**	**UC**	**C** [b]	**C**	**C**	**C**
Storage week	0	4M [c]	4S [d]	4T [e]	12M	12S	12T	24M	24S	24T	0	24M	24S	24T
Hot water extract (%)	33.5	20.3	17.4	14.6	16.4	11.5	8.9	14.3	9.2	7.3	30.6	13.7	5	5.7
Hot water extract SD	0.7	0.9	0.8	1.4	0.3	0.1	0.3	0.2	0.2	1.4	0.2	0.3	0.1	0
Hexane extract (%)	4.1	4.4	4.1	3.8	3.8	4	3.6	4.1	3.4	3	4.3	4.9	3.4	3.6
Hexane extract SD	0.01	0.2	0.001	0.2	0.1	0.1	0.01	0.04	0.04	0.1	0.002	0.03	0.1	0.03
Holocellulose (%)											35.1	34.8	40.4	38.2
Holocellulose SD											0.6	0.8	1.3	0.7
Cellulose (%)	17.2							17	15.7	15.3				
Cellulose SD	0.5							0.4	0.6	0.7				
Hemicellulose (%)	19.2	19.5	17.4	18.8	20.6	21.9	20.3	19.6	22.8	21.2				
Hemicellulose SD	0.3	0.004	0.6	1.5	1.5	2.6	0.8	0.1	0.2	2				
Lignin (acid-insoluble) (%)	16.8							35.8	39.8	44.4	17.4	33.8	37.6	35.9
Lignin (acid-insoluble) SD	0.4							0.4	1.6	2.5	0.8	0.4	1.2	1.3
Lignin (acid-soluble) (%)	0.7							0.7	0.8	0.8	0.7	0.7	1	0.8
Lignin (acid-soluble) SD	0.1							0.04	0.02	0.02	0.02	0.2	0.03	0.04
Unidentified (%)	8.5	55.8	61.1	62.8	59.2	62.7	67.2	8.4	8.3	8	11.9	12.1	12.6	15.7
Unidentified SD	1.7	1.1	1.4	0.04	1	2.8	0.5	0.2	0.5	1.7	0.2	0.4	1.1	1.4

[a] Uncovered [b] Covered with snow [c] Middle [d] Side [e] Top.

Table S2. Values for Figure 3.

**Bark Pile**	**UC** [a]	**UC**	**UC**	**UC**	**UC**	**UC**	**UC**	**UC**	**UC**	**UC**	**C** [b]	**C**	**C**	**C**
Storage week	0	4M [c]	4S [d]	4T [e]	12M	12S	12T	24M	24S	24T	0	24M	24S	24T
Resin acids (mg/g of d.m.)	14.1	10.3	8.6	8.8	8	7.5	9.1	8.5	7.5	8.8	12.2	9.7	9.6	8.1
Resin acids SD	2.2	0.5	0.2	0.1	0.2	0.1	0.03	0.02	0.2	0.3	0.3	0.2	0.2	0.01
Fatty acids (mg/g of d.m.)	9.5	8.1	7.4	7.3	8.7	4.2	4.9	8.4	2.8	2.1	10	8.9	3.4	6.5
Fatty acids SD	0.3	0.2	0.3	0.3	0.1	0.03	0.02	0.1	0.04	0.2	0.03	0.004	0.0004	0.4
Diterpenoids (mg/g of d.m.)	4.1	3.2	2.2	2.3	1.8	2	2.4	1.9	2.1	2.7	3.9	2.4	2.6	2.2
Diterpenoids SD	0.6	0.2	0.04	0.05	0.03	0.02	0.02	0.01	0.1	0.1	0.1	0.1	0.01	0.001
Sterols (mg/g of d.m.)	3.3	4.3	3.2	2.9	3.4	3.6	3.3	2.9	2.7	2.7	3.3	2.9	2.5	2.9
Sterols SD	0.1	0.2	0.2	0.2	0.3	0.3	0.1	0.3	0.1	0.1	0.02	0.1	0.01	0.1
Steryl esters (mg/g of d.m.)	0.6	0.5	0.4	0.4	1	0.5	0.6	1.7	0.5	0.7	1.1	1.5	0.8	0.7
Steryl esters SD	0.1	0.1	0.2	0.1	0.4	0.01	0.1	0.1	0.1	0.1	0.1	0.04	0.1	0.1
Triglycerides (mg/g of d.m.)	2.6	10.9	14.6	11.9	12.5	17.3	12.7	17.6	18.1	12.6	6.4	21.7	14.5	15.6
Triglycerides SD	2.5	0.7	0.7	1.1	0.2	0.5	1.6	0.4	0.7	0.2	0.2	0.2	0.5	0.1
Others (mg/g of d.m.)	2.6	3.6	2.6	2.3	2.5	2.5	2.4	2.1	1.3	0.9	2.7	2.1	1.3	1.9
Others SD	0.3	0.2	0.2	0.2	0.1	0.3	0.01	0.2	0.1	0.1	0.02	0.1	0.02	0.03
Unidentified (mg/g of d.m.)	5.9	4.3	3.4	3.5	2.7	2.1	1.7	1.1	0.3	0.3	5.8	2.3	0.4	0.7
Unidentified SD	0.1	0.001	0.1	0.1	0.02	0.1	0.6	0.1	0.01	0.1	0.2	0.05	0.1	0.03

[a] Uncovered [b] Covered with snow [c] Middle [d] Side [e] Top.

Table S3. Values for Figure 4.

Bark Pile	UC [a]	UC	UC	UC	UC	UC	UC	UC	UC	UC	C [b]	C	C	C
Storage week	0	4M [c]	4S [d]	4T [e]	12M	12S	12T	24M	24S	24T	0	24M	24S	24T
Sugars (mg/g of d.m.)	92.3	21.1	17.9	14.8	30.9	4.8	6.5	44.5	7.8	1.7	82	23.8	1.7	1.9
Sugars SD	9.5	1.9	2.3	0.9	1	0.3	0.7	4.4	0.5	1	10	0.2	0.2	0.04
Sugar alcohols (mg/g of d.m.)	8.1	22	9.3	8.6	6.5	1.6	2.7	10.3	2.2	1.1	8.1	12.7	1.1	0.9
Sugar alcohols SD	0.8	1.7	1	0.1	0.1	0.02	0.3	0.2	0.02	0.5	1.8	0.5	0.1	0.04
Organic acids (mg/g of d.m.)	23.6	15.9	10.6	7.5	16.1	2.9	3.6	25	4.2	1.3	18.8	18.6	1	1.3
Organic acids SD	0.5	1.6	1.4	0.2	0.1	0.1	0.6	0.7	0.1	0.7	0.3	0.2	0.02	0.1
Stilbenes (mg/g of d.m.)	19.4	2.7	0.7	0.7	0.5	0.4	0.4	0.3	0.2	0.2	23	0.3	0.2	0.2
Stilbenes SD	3.4	0.2	0.03	0.1	0.02	0.03	0.001	0.1	0.04	0.1	0.4	0.1	0.02	0.001
Sesquistilbenes (mg/g of d.m.)	16.4	4.3	4.8	3.6	6.1	3.1	4.3	1.1	1.6	0.6	20.9	0	0	0
Sesquistilbenes SD	0.1	0.1	0.5	1.3	0.8	0.9	0.001	0.1	0.5	0.2	2.4	0	0	0
Distilbenes (mg/g of d.m.)	16.9	5.1	4.2	3.8	5.3	3.4	3.9	2.1	2.4	2.1	14.7	0	0	0
Distilbenes SD	0.8	0.6	0.4	0.8	0.2	0.9	0.1	0.9	1.5	0.4	2.2	0	0	0
Flavonoids (mg/g of d.m.)	6.3	2.2	0.9	0.7	0.5	0.3	0.4	0.5	0.03	0.1	5.2	1	0.1	0.1
Flavonoids SD	0.9	0.1	0.1	0.1	0.03	0.02	0.02	0.004	0.04	0.1	1.8	0.2	0.02	0.02
Alcohols (mg/g of d.m.)	3.5	1.2	0.5	0.5	0.6	0.1	0.2	0.5	0.04	0.02	3.4	0.8	0.1	0.05
Alcohols SD	0.5	0.1	0.04	0.05	0.002	0.01	0.01	0.1	0.1	0.03	0.5	0.1	0.01	0.01
Others (mg/g of d.m.)	6.4	8.5	4.1	3.8	4	1.8	2.8	6	4	0.8	6.3	8.7	2	1.9
Others SD	1	0.5	0.5	0.1	0.1	0.1	1.2	2.2	3.4	0.4	0.6	5.2	0.4	0.1
Unidentified (mg/g of d.m.)	141.5	120.1	121.1	101.6	93.3	96.5	64.2	53.1	68.6	64.7	123.8	69.4	44.1	49.7
Unidentified SD	21.7	3.3	13.2	13.6	5.3	3.1	6	6.6	5.2	11.8	20.3	9.5	2.2	0.4

[a] Uncovered [b] Covered with snow [c] Middle [d] Side [e] Top

Table S4. Values for Figure 5.

**Bark Pile**	**UC** [a]	**UC**	**UC**	**UC**	**UC**	**UC**	**UC**	**UC**	**UC**	**UC**	**C** [b]	**C**	**C**	**C**
Storage week	0	4M [c]	4S [d]	4T [e]	12M	12S	12T	24M	24S	24T	0	24M	24S	24T
Dehydroabietic acid (mg/g of d.m.)	2.5	2.1	2.3	2.2	2.6	2.1	2.4	2.6	2.2	2.1	2.1	2.6	2.5	2.4
Dehydroabietic acid SD	0.4	0.1	0.1	0.1	0.1	0.02	0.004	0.01	0.1	0.1	0.1	0.04	0.03	0.1
Isopimaric acid (mg/g of d.m.)	2.1	1.6	1.7	1.7	1.4	1.5	2.3	1.6	1.5	2.7	1.9	1.6	2.7	1.7
Isopimaric acid SD	0.3	0.1	0.05	0.03	0.02	0.01	0.02	0.002	0.03	0.1	0.1	0.1	0.1	0.02
Levopimaric acid (mg/g of d.m.)	1.6	1	0.3	0.3	0.2	0.2	0.2	0.2	0.2	0.1	1.3	0.2	0.2	0.2
Levopimaric acid SD	0.2	0.03	0.01	0.02	0.005	0.001	0.01	0.002	0.01	0.01	0.01	0.03	0.01	0.02
Neoabietic acid (mg/g of d.m.)	1.6	1.2	0.4	0.5	0.2	0.3	0.4	0.2	0.3	0.4	1.3	0.3	0.4	0.3
Neoabietic acid SD	0.3	0.1	0.01	0.01	0.01	0.0003	0.005	0.01	0.01	0.02	0.1	0.1	0.01	0.03
Abietic acid (mg/g of d.m.)	1.5	1.1	1.1	1.2	1	0.8	1	1.1	0.7	0.7	1.3	1.6	0.9	0.9
Abietic acid SD	0.2	0.05	0.02	0.03	0.01	0.004	0.01	0.01	0.02	0.02	0.04	0.1	0.02	0.002
Palustric acid (mg/g of d.m.)	1.1	1.2	0.8	0.8	0.5	0.6	0.6	0.4	0.5	0.4	0.8	0.3	0.4	0.4
Palustric acid SD	0.2	0.1	0.01	0.01	0.01	0.005	0.0005	0.03	0.03	0.004	0.03	0.1	0.03	0.1
Hydroxydehydroabietic acid 1 (mg/g of d.m.)	0.6	0.3	0.3	0.3	0.3	0.3	0.3	0.3	0.3	0.4	0.5	0.5	0.4	0.3
Hydroxydehydroabietic acid 1 SD	0.1	0.01	0.01	0.0002	0.01	0.001	0.003	0.01	0.003	0.01	0.01	0.01	0.01	0.02
Sandaracopimaric acid (mg/g of d.m.)	0.5	0.4	0.3	0.3	0.3	0.3	0.3	0.3	0.3	0.2	0.4	0.4	0.3	0.3
Sandaracopimaric acid SD	0.1	0.02	0.01	0.01	0.01	0.002	0.001	0.001	0.01	0.01	0.01	0.01	0.002	0.002
Hydroxy resin acid (mg/g of d.m.)	0.5	0.1	0.1	0.1	0.1	0.1	0.1	0.2	0.1	0.1	0.6	0.2	0.1	0.1
Hydroxy resin acid SD	0.01	0.01	0.01	0.01	0.01	0.01	0.01	0.004	0.01	0.005	0.01	0.02	0.005	0.01
Hydroxydehydroabietic acid 2 (mg/g of d.m.)	0.4	0.2	0.1	0.1	0.1	0.2	0.1	0	0.2	0.1	0.4	0.2	0.1	0.1
Hydroxydehydroabietic acid 2 SD	0.1	0.005	0.004	0.001	0.01	0.01	0.01	0.005	0.003	0.01	0.02	0.01	0.0001	0.002
Pimaric acid (mg/g of d.m.)	0.3	0.2	0.2	0.2	0.2	0.2	0.3	0.2	0.2	0.3	0.2	0.2	0.2	0.2
Pimaric acid SD	0.03	0.01	0.003	0.01	0.004	0.0003	0.003	0.0005	0.02	0.03	0.04	0.1	0.004	0.004
Cupressic acid (mg/g of d.m.)	0.2	0.1	0.2	0.2	0.2	0.1	0.2	0.2	0.1	0.1	0.2	0.2	0.1	0.2
Cupressic acid SD	0.02	0.01	0.04	0.005	0.001	0.001	0.004	0.001	0.01	0.01	0.004	0.01	0.001	0.01
Imbricatolic acid	0.4	0.3	0.2	0.2	0.2	0.2	0.2	0.1	0.2	0.2	0.5	0.3	0.2	0.2

(mg/g of d.m.)														
Imbricatolic acid SD	0.04	0.01	0.01	0.01	0.004	0.02	0.002	0.000 2	0.01	0.01	0.01	0.003	0.006	0.000 1
7-Oxodehydroabietic acid (mg/g of d.m.)	0.2	0.1	0.1	0.1	0.2	0.1	0.1	0.2	0.1	0.2	0.2	0.2	0.2	0.2
7-Oxodehydroabietic acid SD	0.02	0.003	0.001	0.01	0.01	0.002	0.002	0.005	0.004	0.01	0.004	0.01	0.001	0.004
Secodehydroabietic acid (mg/g of d.m.)	0	0	0.1	0.1	0.1	0.1	0.2	0.1	0.1	0.2	0	0.1	0.2	0.1
Secodehydroabietic acid SD	0.01	0.002	0.005	0.002	0.001	0.000 2	0.001	0.001	0.002	0.01	0.002	0.001	0.01	0.002
4-Hydroxy cinnamic acid (mg/g of d.m.)	0	0.1	0.3	0.3	0.3	0.2	0.4	0.5	0.3	0.5	0.1	0.4	0.5	0.4
4-Hydroxy cinnamic acid SD	0.004	0.01	0.01	0.01	0.003	0.01	0.005	0.01	0.002	0.01	0.002	0.02	0.01	0.01
Other resin acids (mg/g of d.m.)	0.5	0.2	0.2	0.2	0.3	0.2	0.2	0.2	0.2	0.2	0.3	0.4	0.3	0.2
Other resin acids SD	0.1	0.02	0.005	0.03	0.03	0.03	0.001	0.02	0.005	0.01	0.01	0.01	0.01	0.01

[a] Uncovered [b] Covered with snow [c] Middle [d] Side [e] Top.

Table S5. Values for Figure 6.

**Bark Pile**	**UC** [a]	**UC**	**UC**	**UC**	**UC**	**UC**	**UC**	**UC**	**UC**	**UC**	**C** [b]	**C**	**C**	**C**
Storage week	0	4M [c]	4S [d]	4T [e]	12M	12S	12T	24M	24S	24T	0	24M	24S	24T
Acid 18:2 esters (mg/g of d.m.)	2.7	2.2	1.8	1.6	1.6	0.8	0.8	1.5	0.4	0.3	2.8	1.7	0.5	0.8
Acid 18:2 esters SD	0.05	0.1	0.1	0.1	0.02	0.01	0.002	0.1	0.01	0.03	0.005	0.02	0.01	0.1
Acid 18:1 esters (mg/g of d.m.)	2	1.8	1.4	1.3	1.6	0.7	0.6	1	0.2	0.2	2.1	1.1	0.3	0.4
Acid 18:1 esters SD	0.1	0.1	0.1	0.1	0.01	0.01	0.01	0.1	0.01	0.02	0.02	0.04	0.02	0.1
Acid 18:3 esters (mg/g of d.m.)	1.6	1.1	0.9	0.8	0.8	0.3	0.4	0.7	0.2	0.3	1.7	1.1	0.3	0.5
Acid 18:3 esters SD	0.01	0.1	0.1	0.03	0.02	0.003	0.03	0.1	0.1	0.04	0.01	0.02	0.03	0.04
Acid 22:0 esters (mg/g of d.m.)	0.8	0.2	0.2	0.2	0.2	0.2	0.2	0.5	0.3	0.2	0.9	0.6	0.2	0.6
Acid 22:0 esters SD	0.01	0.01	0.02	0.02	0.02	0.002	0.02	0.01	0.03	0.04	0.1	0.01	0.02	0.3
Acid 16:0 esters (mg/g of d.m.)	0.4	0.4	0.3	0.3	0.4	0.2	0.2	0.3	0.1	0.1	0.4	0.3	0.1	0.1
Acid 16:0 esters SD	0.04	0.02	0.02	0.02	0.004	0.001	0.002	0.03	0.002	0.01	0.01	0.01	0.01	0.02
Acid 18:1 (mg/g of d.m.)	0.3	0.4	0.6	0.7	0.7	0.5	0.6	0.7	0.3	0.2	0.2	0.6	0.4	0.9
Acid 18:1 SD	0.1	0.02	0.04	0.03	0.01	0.01	0.02	0.01	0.002	0.01	0.02	0.02	0.01	0.01
Acid 22:0 (mg/g of d.m.)	0.3	0.2	0.2	0.3	0.4	0.2	0.3	0.4	0.2	0.2	0.3	0.4	0.2	0.2
Acid 22:0 SD	0.001	0.003	0.004	0.02	0.01	0.001	0.02	0.01	0.002	0.01	0.004	0.01	0.02	0.0001
Acid 18:3 (mg/g of d.m.)	0.3	0.5	0.5	0.5	0.6	0.3	0.4	0.6	0.3	0.1	0.3	0.6	0.3	0.6
Acid 18:3 SD	0.01	0.01	0.02	0.002	0.002	0.01	0.03	0.01	0.05	0.001	0.01	0.02	0.02	0.01
Acid 17:0 esters (mg/g of d.m.)	0.3	0.2	0.2	0.2	0.2	0.1	0.1	0.1	0.03	0.04	0.3	0.1	0	0
Acid 17:0 esters SD	0.004	0.01	0.01	0.01	0.001	0.001	0.002	0.02	0.002	0.005	0.003	0.01	0.0001	0.01
Acid 18:0 (mg/g of d.m.)	0.2	0.1	0.1	0.1	0.2	0.1	0.1	0.2	0.1	0.1	0.1	0.2	0.2	0.2
Acid 18:0 SD	0.003	0.01	0.01	0.004	0.002	0.00004	0.0003	0.003	0.003	0.004	0.01	0.01	0.004	0.003
Acid 18:2 (mg/g of d.m.)	0.1	0.3	0.4	0.5	0.7	0.3	0.4	0.8	0.2	0.2	0.2	0.7	0.3	0.8
Acid 18:2 SD	0.004	0.001	0.005	0.0002	0.012	0.001	0.02	0.02	0.003	0.003	0.0002	0.02	0.01	0.002
Acid 24:0 esters (mg/g of d.m.)	0.1	0.1	0.2	0.2	0.3	0.2	0.3	0.6	0.2	0.1	0	0.5	0.1	0.6
Acid 24:0 esters	0.02	0.03	0.003	0.01	0.02	0.001	0.02	0.02	0.001	0.02	0	0.02	0.01	0.4

SD														
Acid 24:0 (mg/g of d.m.)	0.2	0.1	0.1	0.1	0.1	0.1	0.1	0.1	0.1	0.1	0.3	0.2	0.1	0.1
Acid 24:0 SD	0.037	0.004	0.002	0.03	0.01	0.002	0.0001	0.005	0.001	0.01	0.01	0.002	0.005	0.004
Acid 16:0 (mg/g of d.m.)	0.1	0.1	0.2	0.2	0.3	0.1	0.2	0.3	0.1	0.1	0.1	0.3	0.1	0.3
Acid 16:0 SD	0.002	0.01	0.01	0.001	0.005	0.002	0.005	0.01	0.001	0.003	0.001	0.01	0.002	0.001
Acid 17:0 (mg/g of d.m.)	0.04	0.1	0.1	0.1	0.1	0.1	0.1	0.1	0.05	0.03	0.04	0.1	0.1	0.2
Acid 17:0 SD	0.02	0.01	0.1	0.1	0.01	0.01	0.05	0.1	0.03	0.02	0.02	0.04	0.03	0.1
2-Hydroxy-24:0 acid (mg/g of d.m.)	0.1	0.1	0.2	0.2	0.3	0.1	0.2	0.4	0.1	0.1	0.1	0.3	0.1	0.2
2-Hydroxy-24:0 acid SD	0.1	0	0.03	0.04	0.01	0.003	0.03	0.01	0.01	0.002	0.005	0.01	0.001	0.001
Other fatty acids (mg/g of d.m.)	0.1	0.03	0.04	0.02	0.03	0.02	0.02	0.1	0.02	0.04	0.1	0.1	0.03	0.04
Other fatty acids SD	0.02	0.01	0.04	0.002	0.02	0.00003	0.0002	0.004	0.01	0.03	0.02	0.001	0.0001	0.002

[a] Uncovered [b] Covered with snow [c] Middle [d] Side [e] Top

Table S6. Values for Figure 7.

**Bark Pile**	**UC** [a]	**UC**	**UC**	**UC**	**UC**	**UC**	**UC**	**UC**	**UC**	**UC**	**C** [b]	**C**	**C**	**C**
Storage week	0	4M [c]	4S [d]	4T [e]	12M	12S	12T	24M	24S	24T	0	24M	24S	24T
Thunbergol (mg/g of d.m.)	1.3	1.0	0.3	0.3	0.1	0.3	0.2	0.1	0.3	0.2	1.2	0.3	0.2	0.3
Thunbergol SD	0.2	0.1	0.001	0.003	0.01	0.03	0.0003	0.01	0.02	0.004	0.03	0.02	0.003	0.01
Δ-13-(*trans*) neoabienol (mg/g of d.m.)	1.3	1.0	0.8	0.8	0.5	0.6	0.6	0.5	0.6	0.5	1.1	0.8	0.6	0.6
Δ-13-(*trans*) neoabienol SD	0.2	0.04	0.02	0.005	0.02	0.01	0.01	0.01	0.02	0.01	0.01	0.01	0.002	0.0004
Cis-abienol (mg/g of d.m.)	0.4	0.2	0.1	0.2	0.1	0.1	0.1	0.1	0.1	0.2	0.4	0.2	0.1	0.2
Cis-abienol SD	0.04	0.01	0.004	0.01	0.004	0.01	0.01	0.003	0.001	0.01	0.02	0.01	0.001	0.001
Isopimaral (mg/g of d.m.)	0.2	0.1	0.1	0.1	0.1	0.1	0.1	0.2	0.2	0.2	0.2	0.2	0.2	0.1
Isopimaral SD	0.1	0.01	0.004	0.01	0.002	0.01	0.01	0.003	0.05	0.01	0.001	0.02	0.01	0.01
Palustral (mg/g of d.m.)	0.2	0.2	0.2	0.2	0.1	0.1	0.2	0.1	0.1	0.2	0.1	0.1	0.2	0.1
Plaustral SD	0.02	0.01	0.003	0.01	0.003	0.0002	0.001	0.004	0.01	0.004	0.01	0.01	0.01	0.01
Isopimarol (mg/g of d.m.)	0.1	0.1	0.1	0.1	0.1	0.1	0.1	0.1	0.1	0.1	0.1	0.1	0.1	0.1
Isopimarol SD	0.1	0.01	0.004	0.01	0.002	0.01	0.01	0.003	0.05	0.01	0.001	0.02	0.01	0.01
Dehydroabietal (mg/g of d.m.)	0.1	0.1	0.1	0.1	0.1	0.1	0.1	0.1	0.1	0.1	0.1	0.1	0.1	0.1
Dehydroabietal SD	0.02	0.01	0.005	0.004	0.003	0.001	0.0004	0.001	0.003	0.001	0.01	0.003	0.00002	0.001
Pimaradiene (mg/g of d.m.)	0.1	0.1	0.1	0.1	0.1	0.1	0.1	0.1	0.1	0.1	0.1	0.1	0.1	0.1
Pimaradiene SD	0.02	0.01	0.003	0.004	0.004	0.002	0.001	0.002	0.0002	0.003	0.002	0.001	0.002	0.0002
Manool (mg/g of d.m.)	0.1	0.1	0.1	0.1	0.1	0.1	0.1	0.1	0.1	0.1	0.1	0.1	0.2	0.1
Manool SD	0.02	0.02	0.005	0.01	0.003	0.001	0.003	0.001	0.001	0.01	0.001	0.001	0.001	0.001
Epimanoyl oxide (mg/g of d.m.)	0.1	0.1	0.1	0.1	0.1	0.1	0.1	0.2	0.1	0.1	0.1	0.1	0.1	0.1
Epimanoyl oxide SD	0.01	0.01	0.01	0.0003	0.001	0.001	0.002	0.001	0.001	0.003	0.0002	0.02	0.001	0.001
Abietal (mg/g of d.m.)	0.04	0.03	0.03	0.03	0.03	0.01	0.03	0.04	0.02	0.00	0.04	0.05	0.03	0.02
Abietal SD	0.003	0.002	0.002	0.0002	0.001	0.01	0.001	0.0001	0.002	0.0	0.001	0.003	0.00002	0.001
Thunbergene (mg/g of d.m.)	0.05	0.04	0.04	0.04	0.05	0.04	0.04	0.04	0.04	0.04	0.1	0.1	0.03	0.05
Thunbergene	0.01	0.01	0.002	0.01	0.003	0.001	0.001	0.002	0.01	0.004	0.01	0.001	0.01	0.003

SD														
Methyl-8,15-isopimaradien-18-oate (mg/g of d.m.)	0.1	0.0	0.1	0.1	0.1	0.2	0.4	0.1	0.2	0.7	0.04	0.04	0.6	0.2
Methyl-8,15-isopimaradien-18-oate SD	0.003	0.002	0.002	0.0002	0.001	0.01	0.001	0.0001	0.002	0.0	0.001	0.003	0.00002	0.001
Methyl neoabietate (mg/g of d.m.)	0.03	0.02	0.03	0.03	0.01	0.02	0.02	0.01	0.00	0.02	0.02	0.00	0.02	0.01
Methyl neoabietate SD	0.01	0.001	0.003	0.001	0.01	0.001	0.0003	0.0003	0.0	0.000	0.003	0.0	0.0001	0.003
Vanillin (mg/g of d.m.)	0.01	0.04	0.03	0.1	0.05	0.03	0.04	0.1	0.04	0.05	0.05	0.1	0.1	0.03
Vanillin SD	0.01	0.02	0.01	0.002	0.005	0.003	0.002	0.01	0.002	0.003	0.01	0.004	0.003	0.002

[a] Uncovered [b] Covered with snow [c] Middle [d] Side [e] Top

Table S7. Values for Figure 8.

**Bark Pile**	**UC** [a]	**UC**	**UC**	**UC**	**UC**	**UC**	**UC**	**UC**	**UC**	**UC**	**C** [b]	**C**	**C**	**C**
Storage week	0	4M [c]	4S [d]	4T [e]	12M	12S	12T	24M	24S	24T	0	24M	24S	24T
Sitosterol esters (mg/g of d.m.)	1.8	1.6	1.4	1.3	1.0	1.0	0.9	0.8	0.4	0.2	0.9	0.9	0.7	0.8
Sitosterol esters SD	0.1	0.04	0.2	0.1	0.02	0.04	0.01	0.02	0.1	0.02	0.1	0.01	0.04	0.02
Other steryl esters (mg/g of d.m.)	0.7	1.7	0.9	0.7	1.2	1.2	1.2	1.1	0.8	0.7	1.6	1.0	0.5	0.8
Other steryl esters SD	0.1	0.1	0.003	0.1	0.2	0.3	0.03	0.2	0.02	0.03	0.1	0.1	0.04	0.1
Campesterol esters (mg/g of d.m.)	0.4	0.3	0.3	0.3	0.2	0.2	0.2	0.2	0.1	0.0	0.2	0.2	0.1	0.2
Campesterol esters SD	0.02	0.01	0.04	0.02	0.02	0.01	0.01	0.02	0.02	0.01	0.02	0.001	0.01	0.1
Sitosterol (mg/g of d.m.)	0.3	0.4	0.4	0.4	0.5	0.6	0.5	0.5	0.7	0.8	0.4	0.5	0.6	0.6
Sitosterol SD	0.02	0.02	0.02	0.03	0.03	0.002	0.01	0.02	0.003	0.01	0.01	0.01	0.001	0.002
Campesterol (mg/g of d.m.)	0.1	0.1	0.1	0.1	0.1	0.1	0.1	0.1	0.1	0.2	0.1	0.1	0.1	0.1
Campesterol SD	0.005	0.003	0.001	0.004	0.02	0.001	0.003	0.003	0.002	0.001	0.001	0.01	0.002	0.01
24-Methylenecycloartan-3-one (mg/g of d.m.)	0.04	0.04	0.05	0.1	0.1	0.1	0.1	0.0	0.1	0.1	0.04	0.1	0.1	0.1
24-Methylenecycloartan-3-one SD	0.001	0.01	0.0004	0.001	0.001	0.002	0.003	0.003	0.002	0.002	0.0001	0.003	0.003	0.001
7-Hydroxysistosterol (mg/g of d.m.)	0.02	0.1	0.02	0.03	0.1	0.2	0.1	0.1	0.2	0.3	0.1	0.1	0.2	0.1
7-Hydroxysistosterol SD	0.005	0.001	0.0002	0.005	0.04	0.000004	0.03	0.0004	0.03	0.003	0.001	0.01	0.01	0.04
Chondrillasterol (mg/g of d.m.)	0.0	0.01	0.02	0.02	0.1	0.1	0.1	0.1	0.3	0.3	0.02	0.1	0.2	0.1
Chondrillasterol SD	0.0	0.01	0.004	0.01	0.03	0.01	0.02	0.02	0.01	0.003	0.003	0.001	0.004	0.01
Ergosterol (mg/g of d.m.)	0.02	0.02	0.02	0.01	0.03	0.04	0.03	0.01	0.04	0.03	0.02	0.02	0.02	0.01
Ergosterol SD	0.02	0.001	0.004	0.001	0.02	0.0001	0.01	0.001	0.002	0.002	0.0003	0.002	0.01	0.02

[a] Uncovered [b] Covered with snow [c] Middle [d] Side [e] Top

Table S8. Values for Figure 9.

Bark Pile	UC [a]	UC	UC	UC	UC	UC	UC	UC	UC	UC	C [b]	C	C	C
Storage week	0	4M [c]	4S [d]	4T [e]	12M	12S	12T	24M	24S	24T	0	24M	24S	24T
Glucose (mg/g of d.m.)	51.1	12.0	9.6	7.0	16.6	1.6	2.3	20.2	3.9	0.3	43.8	1.3	0.1	0.1
Glucose SD	3.2	1.2	1.4	0.8	0.4	0.2	0.3	1.5	0.01	0.1	3.2	0.3	0.01	0.01
Sucrose (mg/g of d.m.)	27.3	1.1	0.8	2.1	0.8	0.2	0.1	1.1	0.2	0.1	27.8	0.3	0.2	0.2
Sucrose SD	4.0	0.2	0.02	0.1	0.4	0.01	0.05	0.1	0.1	0.1	4.0	0.1	0.02	0.01
Maltose (mg/g of d.m.)	5.2	1.8	0.7	0.7	0.9	0.5	0.6	1.1	0.5	0.4	4.5	1.0	0.5	0.5
Maltose SD	0.6	0.2	0.1	0.02	0.1	0.01	0.01	0.3	0.05	0.2	0.6	0.2	0.1	0.004
Alpha lactose (mg/g of d.m.)	2.7	0.7	0.6	0.3	0.2	0.1	0.05	0.1	0.1	0.03	2.4	0.1	0.05	0.04
Alpha lactose SD	0.4	0.1	0.1	0.003	0.0002	0.03	0.01	0.1	0.1	0.04	0.3	0.02	0.01	0.001
Galactose (mg/g of d.m.)	2.0	1.9	2.5	2.7	10.3	1.7	2.7	19.1	2.4	0.5	1.2	17.0	0.5	0.6
Galactose SD	0.6	0.2	0.3	0.1	0.2	0.1	0.4	2.2	0.2	0.3	0.4	0.7	0.03	0.01
Trehalose (mg/g of d.m.)	1.6	0.6	0.8	0.6	0.3	0.2	0.1	0.2	0.3	0.1	1.1	0.2	0.2	0.1
Trehalose SD	0.3	0.02	0.1	0.01	0.03	0.01	0.002	0.1	0.1	0.2	0.1	0.1	0.03	0.001
Palatinose (mg/g of d.m.)	1.1	0.2	0.1	0.1	0.0	0.0	0.0	0.0	0.0	0.0	0.7	0.0	0.0	0.0
Palatinose SD	0.1	0.01	0.01	0.001	0.0	0.0	0.0	0.0	0.0	0.0	0.1	0.0	0.0	0.0
Cellobiose (mg/g of d.m.)	0.7	0.1	0.1	0.1	0.2	0.1	0.0	0.2	0.04	0.03	0.4	0.1	0.03	0.04
Cellobiose SD	0.3	0.002	0.03	0.0001	0.003	0.004	0.0	0.03	0.1	0.04	0.01	0.02	0.004	0.0005
Lactulose (mg/g of d.m.)	0.4	0.3	0.2	0.1	0.1	0.1	0.1	0.1	0.1	0.0	0.4	0.1	0.01	0.0
Lactulose SD	0.1	0.02	0.003	0.003	0.01	0.01	0.01	0.01	0.01	0.0	0.1	0.01	0.001	0.0
Glucose phosphate (mg/g of d.m.)	0.2	0.6	0.5	0.1	0.2	0.1	0.1	0.2	0.02	0.1	0.5	0.4	0.1	0.1
Glucose phosphate SD	0.3	0.1	0.1	0.01	0.002	0.01	0.01	0.002	0.04	0.01	0.04	0.1	0.004	0.003
Mannose (mg/g of d.m.)	0.0	1.8	2.1	1.0	1.5	0.4	0.5	2.3	0.4	0.1	0.0	3.4	0.1	0.1
Mannose SD	0.0	0.2	0.3	0.05	0.03	0.01	0.1	0.2	0.02	0.02	0.0	0.1	0.04	0.01

[a] Uncovered [b] Covered with snow [c] Middle [d] Side [e] Top

Table S9. Values for Figure 10.

**Bark Pile**	**UC** [a]	**UC**	**UC**	**UC**	**UC**	**UC**	**UC**	**UC**	**UC**	**UC**	**C** [b]	**C**	**C**	**C**
Storage week	0	4M [c]	4S [d]	4T [e]	12M	12S	12T	24M	24S	24T	0	24M	24S	24T
Pinitol (mg/g of d.m.)	5.0	4.8	1.9	1.6	2.0	0.4	0.7	3.4	0.4	0.1	4.8	4.6	0.2	0.1
Pinitol SD	0.8	0.4	0.2	0.03	0.05	0.01	0.02	0.2	0.02	0.03	0.7	0.01	0.03	0.01
Maltotriitol (mg/g of d.m.)	1.0	0.1	0.1	0.1	0.1	0.1	0.1	0.1	0.0	0.0	1.3	0.2	0.2	0.1
Maltotriitol SD	0.04	0.002	0.01	0.001	0.02	0.002	0.003	0.1	0.0	0.0	0.4	0.04	0.01	0.004
Inositol (mg/g of d.m.)	0.6	6.0	1.9	1.2	0.4	0.1	0.1	0.6	0.1	0.05	0.5	0.5	0.02	0.02
Inositol SD	0.02	0.4	0.02	0.1	0.0001	0.004	0.005	0.03	0.001	0.002	0.1	0.02	0.001	0.001
Sorbitol (mg/g of d.m.)	0.4	1.4	0.4	0.5	0.5	0.1	0.2	0.7	0.2	0.1	0.4	1.2	0.1	0.04
Sorbitol SD	0.03	0.1	0.1	0.01	0.03	0.003	0.004	0.02	0.02	0.02	0.1	0.1	0.001	0.003
Arabitol (mg/g of d.m.)	0.4	1.9	0.8	0.7	1.4	0.2	0.4	2.3	0.5	0.3	0.7	1.6	0.3	0.2
Arabitol SD	0.02	0.2	0.1	0.03	0.002	0.02	0.1	0.1	0.1	0.1	0.5	0.01	0.003	0.01
Isomaltitol (mg/g of d.m.)	0.3	0.1	0.1	0.0	0.0	0.0	0.1	0.1	0.0	0.1	0.3	0.1	0.1	0.1
Isomaltitol SD	0.04	0.0001	0.004	0.0	0.0	0.0	0.02	0.01	0.0	0.02	0.1	0.2	0.01	0.01
Mannitol (mg/g of d.m.)	0.3	6.6	3.1	3.5	1.0	0.4	0.5	1.5	0.5	0.2	0.2	2.6	0.05	0.03
Mannitol SD	0.03	0.6	0.5	0.2	0.02	0.01	0.002	0.1	0.03	0.1	0.02	0.2	0.01	0.001
L-Ribulose (mg/g of d.m.)	0.1	0.7	0.6	0.5	0.6	0.2	0.3	1.0	0.3	0.3	0.0	1.5	0.2	0.2
L-Ribulose SD	0.1	0.1	0.1	0.01	0.01	0.005	0.04	0.02	0.02	0.2	0.0	0.05	0.002	0.02
Erythritol (mg/g of d.m.)	0.0	0.2	0.4	0.4	0.5	0.2	0.3	0.7	0.3	0.0	0.0	0.6	0.03	0.04
Erythritol SD	0.0	0.02	0.1	0.1	0.1	0.01	0.1	0.2	0.1	0.0	0.0	0.3	0.001	0.006
Maltitol (mg/g of d.m.)	0.0	0.0	0.0	0.0	0.0	0.0	0.0	0.0	0.0	0.0	0.0	0.04	0.01	0.02
Maltitol SD	0.0	0.0	0.0	0.0	0.0	0.0	0.0	0.0	0.0	0.0	0.0	0.02	0.001	0.02

[a] Uncovered [b] Covered with snow [c] Middle [d] Side [e] Top

Table S10. Values for Figure 11.

**Bark Pile**	**UC** [a]	**UC**	**UC**	**UC**	**UC**	**UC**	**UC**	**UC**	**UC**	**UC**	**C** [b]	**C**	**C**	**C**
Storage week	0	4M [c]	4S [d]	4T [e]	12M	12S	12T	24M	24S	24T	0	24M	24S	24T
Gluconic acid (mg/g of d.m.)	7.0	6.9	3.3	2.8	5.0	1.1	1.4	7.2	1.6	0.5	5.4	6.1	0.4	0.6
Gluconic acid SD	0.7	0.8	0.6	0.1	0.1	0.02	0.1	0.5	0.1	0.1	0.4	0.4	0.005	0.04
Citric acid (mg/g of d.m.)	6.7	1.4	1.7	0.7	0.7	0.1	0.2	1.0	0.2	0.04	5.0	0.4	0.02	0.02
Citric acid SD	0.8	0.1	0.3	0.1	0.02	0.001	0.01	0.01	0.02	0.1	0.3	0.1	0.001	0.002
Quinic acid (mg/g of d.m.)	7.1	3.6	1.7	1.0	1.9	0.1	0.3	2.8	0.3	0.0	6.3	0.9	0.003	0.0
Quinic acid SD	0.8	0.3	0.1	0.1	0.1	0.0003	0.1	0.2	0.004	0.0	0.1	0.04	0.005	0.0
Shikimic acid (mg/g of d.m.)	1.1	1.5	0.4	0.4	1.1	0.2	0.3	1.0	0.4	0.1	1.1	1.0	0.05	0.1
Shikimic acid SD	0.02	0.2	0.1	0.004	0.4	0.001	0.02	0.03	0.02	0.1	0.04	0.01	0.004	0.004
Malic acid (mg/g of d.m.)	0.8	0.4	0.2	0.1	0.2	0.0	0.0	0.4	0.03	0.05	0.7	0.2	0.04	0.1
Malic acid SD	0.1	0.1	0.02	0.0003	0.03	0.0	0.0	0.03	0.05	0.1	0.1	0.02	0.003	0.01
L-Glutamic acid (mg/g of d.m.)	0.3	1.2	1.5	1.2	5.9	0.8	1.0	11.9	1.4	0.4	0.3	8.0	0.1	0.1
L-Glutamic acid SD	0.1	0.1	0.2	0.1	0.4	0.03	0.3	1.4	0.02	0.4	0.2	0.3	0.002	0.01
2,3-Dihydroxypropanoic acid (mg/g of d.m.)	0.4	0.1	0.3	0.3	0.3	0.3	0.2	0.3	0.1	0.1	0.0	0.4	0.1	0.2
2,3-Dihydroxypropanoic acid SD	0.3	0.02	0.04	0.01	0.01	0.01	0.02	0.02	0.002	0.005	0.0	0.04	0.005	0.1
2,3-Dihydroxysuccinic acid (mg/g of d.m.)	0.0	0.1	0.2	0.3	0.4	0.1	0.1	0.6	0.1	0.03	0.0	0.4	0.02	0.02
2,3-Dihydroxysuccinic acid SD	0.0	0.01	0.03	0.01	0.01	0.002	0.03	0.003	0.001	0.04	0.0	0.04	0.001	0.002
Other (mg/g of d.m.)	0.2	0.7	0.9	0.7	0.6	0.2	0.1	0.8	0.1	0.1	0.0	1.2	0.3	0.2
Other SD	0.01	0.04	0.04	0.1	0.02	0.003	0.03	0.01	0.03	0.1	0.0	0.02	0.02	0.01

[a] Uncovered [b] Covered with snow [c] Middle [d] Side [e] Top

Table S11. Values for Figure 12.

**Bark Pile**	**UC** [a]	**UC**	**UC**	**UC**	**UC**	**UC**	**UC**	**UC**	**UC**	**UC**	**C** [b]	**C**	**C**	**C**
Storage week	0	4M [c]	4S [d]	4T [e]	12M	12S	12T	24M	24S	24T	0	24M	24S	24T
Distilbenes (mg/g of d.m.)	16.9	5.1	4.2	3.8	5.3	3.4	3.9	2.1	2.4	2.1	14.7	0.0	0.0	0.0
Distilbenes SD	0.8	0.6	0.4	0.8	0.2	0.9	0.1	0.9	1.5	0.4	2.2	0.0	0.0	0.0
Sesquistilbenes (mg/g of d.m.)	16.4	4.3	4.8	3.6	6.1	3.1	4.3	1.1	1.6	0.6	20.9	0.0	0.0	0.0
Sesquistilbenes SD	0.1	0.1	0.5	1.3	0.8	0.9	0.001	0.1	0.5	0.2	2.4	0.0	0.0	0.0
Isorhapontin (mg/g of d.m.)	9.5	0.2	0.1	0.05	0.04	0.1	0.03	0.0	0.0	0.0	11.4	0.0	0.0	0.0
Isorhapontin SD	1.7	0.1	0.003	0.003	0.001	0.0004	0.001	0.0	0.0	0.0	0.4	0.0	0.0	0.0
Astringin (mg/g of d.m.)	4.8	0.1	0.1	0.04	0.05	0.1	0.03	0.0	0.0	0.0	5.2	0.0	0.0	0.0
Astringin SD	1.1	0.1	0.1	0.01	0.005	0.01	0.003	0.0	0.0	0.0	0.3	0.0	0.0	0.0
Piceid (mg/g of d.m.)	3.2	0.1	0.1	0.05	0.1	0.04	0.04	0.0	0.0	0.0	4.3	0.0	0.0	0.0
Piceid SD	0.7	0.1	0.01	0.01	0.002	0.004	0.002	0.0	0.0	0.0	0.2	0.0	0.0	0.0
Rhapontigenin (mg/g of d.m.)	1.3	0.5	0.1	0.1	0.1	0.1	0.04	0.02	0.02	0.02	1.6	0.05	0.04	0.04
Rhapontigenin SD	0.2	0.02	0.001	0.001	0.005	0.01	0.002	0.03	0.03	0.03	0.4	0.02	0.01	0.003
Piceatannol (mg/g of d.m.)	0.4	0.9	0.3	0.4	0.1	0.1	0.1	0.2	0.1	0.1	0.3	0.1	0.05	0.1
Piceatannol SD	0.03	0.04	0.03	0.03	0.003	0.01	0.001	0.05	0.004	0.1	0.1	0.03	0.003	0.0002
Resveratrol (mg/g of d.m.)	0.2	0.9	0.1	0.1	0.1	0.1	0.1	0.1	0.1	0.04	0.2	0.1	0.1	0.1
Resveratrol SD	0.01	0.1	0.01	0.005	0.002	0.01	0.0003	0.02	0.01	0.1	0.02	0.02	0.01	0.003

[a] Uncovered [b] Covered with snow [c] Middle [d] Side [e] Top

Table S12. Values for Figure 13.

**Bark Pile**	**UC** [a]	**UC**	**UC**	**UC**	**UC**	**UC**	**UC**	**UC**	**UC**	**UC**	**C** [b]	**C**	**C**	**C**
Storage week	0	4M [c]	4S [d]	4T [e]	12M	12S	12T	24M	24S	24T	0	24M	24S	24T
Taxifolin glycoside (mg/g of d.m.)	1.5	0.2	0.1	0.1	0.0	0.0	0.0	0.0	0.0	0.0	1.5	0.0	0.0	0.0
Taxifolin glycoside SD	0.05	0.04	0.01	0.003	0.0	0.0	0.0	0.0	0.0	0.0	0.7	0.0	0.0	0.0
Naringin (mg/g of d.m.)	1.4	0.5	0.1	0.1	0.0	0.0	0.0	0.0	0.0	0.0	1.3	0.0	0.0	0.0
Naringin SD	0.5	0.03	0.1	0.001	0.0	0.0	0.0	0.0	0.0	0.0	0.3	0.0	0.0	0.0
Catechin (mg/g of d.m.)	1.1	0.3	0.0	0.1	0.03	0.0	0.0	0.0	0.0	0.0	1.0	0.04	0.02	0.02
Catechin SD	0.1	0.04	0.0	0.003	0.04	0.0	0.0	0.0	0.0	0.0	0.2	0.02	0.002	0.0002
Taxifolin (mg/g of d.m.)	1.0	0.2	0.1	0.1	0.1	0.1	0.1	0.1	0.0	0.0	0.9	0.2	0.04	0.05
Taxifolin SD	0.2	0.01	0.003	0.001	0.002	0.0002	0.01	0.04	0.0	0.0	0.2	0.02	0.003	0.004
Neohesperidin (mg/g of d.m.)	0.7	0.0	0.0	0.0	0.0	0.0	0.0	0.0	0.0	0.0	0.3	0.0	0.0	0.0
Neohesperidin SD	0.02	0.0	0.0	0.0	0.0	0.0	0.0	0.0	0.0	0.0	0.5	0.0	0.0	0.0
Naringenin chal-cone (mg/g of d.m.)	0.3	0.4	0.4	0.2	0.1	0.1	0.2	0.1	0.0	0.0	0.0	0.3	0.03	0.02
Naringenin chal-cone SD	0.03	0.02	0.02	0.1	0.0004	0.01	0.03	0.01	0.0	0.0	0.0	0.1	0.004	0.004
Dihydromyricetin (mg/g of d.m.)	0.3	0.6	0.2	0.2	0.2	0.1	0.1	0.2	0.03	0.1	0.1	0.5	0.04	0.1
Dihydromyricetin SD	0.03	0.1	0.02	0.001	0.01	0.01	0.01	0.1	0.04	0.1	0.1	0.1	0.01	0.01
Luteolin (mg/g of d.m.)	0.0	0.0	0.0	0.1	0.04	0.0	0.0	0.0	0.0	0.0	0.0	0.0	0.0	0.0
Luteolin SD	0.0	0.0	0.0	0.0003	0.003	0.0	0.0	0.0	0.0	0.0	0.0	0.0	0.0	0.0

[a] Uncovered [b] Covered with snow [c] Middle [d] Side [e] Top

Table S13. Values for Figure 14.

**Bark Pile**	**UC** [a]	**UC**	**UC**	**UC**	**UC**	**UC**	**UC**	**UC**	**UC**	**UC**	**C** [b]	**C**	**C**	**C**
Storage week	0	4M [c]	4S [d]	4T [e]	12M	12S	12T	24M	24S	24T	0	24M	24S	24T
Procyanidins (g/100g of d.m.)	2.8	1.3	0.6	0.5	0.4	0.4	0.3	0.3	0.3	0.2	2.9	0.5	0.2	0.3
Procyanidins SD	0.2	0.1	0.03	0.04	0.02	0.04	0.03	0.02	0.03	0.02	0.1	0.05	0.02	0.02
Prodelphinidins (g/100g of d.m.)	0.2	0.1	0.1	0.1	0.1	0.1	0.1	0.1	0.04	0.03	0.3	0.1	0.04	0.04
Prodelphinidins SD	0.02	0.01	0.01	0.003	0.01	0.01	0.01	0.005	0.004	0.003	0.01	0.01	0.002	0.003
Degree of polymerisation	8.0	5.7	2.8	2.6	2.4	2.5	2.4	2.2	2.1	2.3	8.2	2.9	2.6	3.0
Degree of polymerisation SD	0.2	0.3	0.1	0.1	0.1	0.2	0.1	0.1	0.1	0.1	0.3	0.2	0.2	0.3

[a] Uncovered [b] Covered with snow [c] Middle [d] Side [e] Top

Table S14. Values for Figure 15.

**Bark Pile**	**UC** [a]	**UC**	**UC**	**UC**	**C** [b]	**C**	**C**	**C**
Storage week	0	24M [c]	24S [d]	24T [e]	0	24M	24S	24T
Glucose (% of d.m.)	38.5	34.1	31.8	29.9	35.3	31.2	30.0	30.7
Glucose SD	0.6	0.5	0.6	0.6	0.6	0.7	1.0	0.6
Arabinose (% of d.m.)	7.8	1.6	2.9	2.3	7.6	3.3	2.5	3.6
Arabinose SD	0.2	0.02	0.02	0.1	0.1	0.1	0.1	0.1
Xylose (% of d.m.)	4.8	3.8	3.7	3.5	4.4	3.5	3.1	3.2
Xylose SD	0.2	0.2	0.1	0.1	0.2	0.1	0.1	0.1
Mannose (% of d.m.)	4.3	3.7	4.0	3.3	4.2	2.7	2.7	2.9
Mannose SD	0.1	0.3	0.1	0.1	0.3	0.1	0.1	0.1
Galactose (% of d.m.)	2.8	1.7	1.8	1.8	2.5	2.0	1.7	1.8
Galactose SD	0.05	0.02	0.05	0.1	0.05	0.1	0.1	0.05

[a] Uncovered [b] Covered with snow [c] Middle [d] Side [e] Top

Table S15. Values for Figure 16.

Bark Pile	UC [a]	UC	UC	UC	UC	UC	UC	UC	UC	UC
Storage week	0	4M [c]	4S [d]	4T [e]	12M	12S	12T	24M	24S	24T
Galacturonic acid (mg/g of d.m.)	93.9	86.9	83.1	80.8	79.8	59.4	42.2	43.6	42.9	44.0
Galacturonic acid SD	7.3	12.0	2.3	0.5	8.5	26.5	3.2	0.04	5.0	2.0
Arabinose (mg/g of d.m.)	69.4	54.1	32.9	31.3	28.1	28.3	44.0	23.4	29.6	37.6
Arabinose SD	0.4	0.2	2.0	0.3	6.9	5.3	8.3	1.3	4.0	0.2
Xylose (mg/g of d.m.)	39.2	30.3	29.3	29.1	44.4	41.3	49.4	52.6	48.0	50.9
Xylose SD	1.8	4.7	4.7	0.6	5.8	9.5	2.7	0.6	4.2	0.9
Galactose (mg/g of d.m.)	38.2	28.4	20.0	18.8	22.6	25.6	34.1	33.3	31.7	34.0
Galactose SD	0.8	4.7	1.3	1.3	3.1	9.0	3.5	1.4	3.4	0.4
Glucose (mg/g of d.m.)	30.0	31.9	39.1	38.3	49.0	45.2	46.2	53.5	54.2	54.3
Glucose SD	0.6	1.1	3.5	4.4	6.1	7.7	3.3	1.8	4.7	0.9
Mannose (mg/g of d.m.)	23.0	16.7	19.1	16.2	23.5	25.4	28.8	26.7	22.5	31.8
Mannose SD	3.3	1.6	5.1	3.2	1.0	5.0	1.2	1.4	1.0	5.0
Rhamnose (mg/g of d.m.)	9.4	7.7	5.7	6.0	7.8	5.5	7.2	5.4	6.0	6.8
Rhamnose SD	0.2	0.1	0.4	0.7	2.3	0.9	1.3	0.2	0.9	0.2
Glucuronic acid (mg/g of d.m.)	4.0	3.3	1.1	1.0	2.6	1.5	6.6	1.9	1.6	1.3
Glucuronic acid SD	0.9	0.04	0.4	0.3	1.8	0.1	7.3	1.0	0.3	0.3

[a] Uncovered [b] Covered with snow [c] Middle [d] Side [e] Top
